# Structure Motivator: A tool for exploring small three-dimensional elements in proteins

**DOI:** 10.1186/1472-6807-12-26

**Published:** 2012-10-16

**Authors:** David P Leader, E James Milner-White

**Affiliations:** 1 College of Medical, Veterinary and Life Sciences, University of Glasgow, Glasgow, G12 8QQ, UK

**Keywords:** Protein motif, Ramachandran plot, Dihedral angle, Relational database

## Abstract

**Background:**

Protein structures incorporate characteristic three-dimensional elements defined by some or all of hydrogen bonding, dihedral angles and amino acid sequence. The software application, Structure Motivator, allows interactive exploration and analysis of such elements, and their resolution into sub-classes.

**Results:**

Structure Motivator is a standalone application with an embedded relational database of proteins that, as a starting point, can furnish the user with a palette of unclassified small peptides or a choice of pre-classified structural motifs. Alternatively the application accepts files of data generated externally. After loading, the structural elements are displayed as two-dimensional plots of dihedral angles (φ/ψ, φ/χ1 or in combination) for each residue, with visualization options to allow the conformation or amino acid composition at one residue to be viewed in the context of that at other residues. Interactive selections may then be made and structural subsets saved to file for further sub-classification or external analysis. The application has been applied both to classical motifs, such as the β-turn, and ‘non-motif’ structural elements, such as specific segments of helices.

**Conclusions:**

Structure Motivator allows structural biologists, whether or not they possess computational skills, to subject small structural elements in proteins to rapid interactive analysis that would otherwise require complex programming or database queries. Within a broad group of structural motifs, it facilitates the identification and separation of sub-classes with distinct stereochemical properties.

## Background

Comparing the architecture of different proteins can provide insights into the principles of their formation and function. Where proteins are very similar it can be useful to superimpose and inspect their three-dimensional structures computationally [1]. Proteins with less overall similarity may still share a common arrangement of secondary-structure features, as exemplified by the CATH [2] and SCOP [3] classification schemata. Outside the regions of secondary structure one can identify smaller structural motifs such as the β-turn [4], generally ranging from three to six residues in length, and defined by specific residues having particular dihedral angles or arrangements of hydrogen bonds [5]. In our studies of such small structural motifs, we needed to compare and analyse them, and separate the sub-classes that they often encompass. It was for this purpose that we developed Structure Motivator, the software described here.

Small structural elements in proteins are recognized by visual inspection of individual proteins using programs that display three-dimensional graphics, and they may then be compared by superimposition in programs of the same type (e.g. Figure nine of [6]). However superimposition is not a practicable means of comparison for large sets of small structural motifs (hundreds and more). The most common practice is to display two-dimensional plots of the φ and ψ dihedral angles (Ramachandran plots [7]) at each residue [8-16]. Our program, Structure Motivator, also employs plots of dihedral angles of small three-dimensional structural elements. However, rather than merely providing a visualization end-point, the plots serve as a starting point for the interactive exploration of such elements.

Structure Motivator will be of value to structural biologists wishing to analyse existing small structural elements in proteins, sub-classify them, and define new ones. It allows users with no knowledge of relational databases to make what are, in effect, complex database queries defining new structural motifs, merely by selecting areas of the plots using a computer mouse.

## Implementation

Structure Motivator has been designed for the desktop — rather than as a web application — to facilitate data input and output, to allow graphics to be saved and printed easily, and so that it can be used in the absence of an internet connection. It was written in the Java programming language so that it could be deployed across different platforms, and gratuitous version inflation was avoided for the benefit of those using older computers.

The Protein Motif Database [5], implemented in the MySQL database management system, was used during development and for preparing input files distributed with the application. A modified version of this was prepared (see Additional file 1, for the schema) and migrated using DdlUtils [17] to the Derby database management system [18], which is written in Java and may be embedded in Java applications. Both Structure Motivator and the PreMotivator utility contain this embedded database.

To optimize performance, SQL (Structured Query Language) queries to the embedded database in Structure Motivator are only used for the initial creation of Java objects corresponding to a chosen motif. Subsequent selections are made by addressing these objects in memory. SQL queries employing JDBC (Java Database Connectivity) were needed to prepare files for use in Structure Motivator, and this functionality was provided in a separate utility, PreMotivator. The format for Structure Motivator text files was chosen to facilitate conversion from tables resulting from SQL queries.

Structure Motivator provides one option that does require an internet connection. It can link to a web facility that allows individual motifs to be viewed in the context of the three-dimensional structure of the protein. This uses a server-based Perl CGI (Common Gateway Interface) application, motivator.cgi, to return a web page with appropriate three-dimensional visualization of the motif with the Jmol applet [19] and additional JavaScript controls. The server is that used for the Motivated Proteins web application which houses both the MySQL database corresponding to that in Structure Motivator and the set of 429 curated files of protein subunits [13], both of which are used by the CGI application. However the JavaScript/Live Connect controls provided for the user differ from those in Motivated Proteins, being tailored to the requirements of Structure Motivator.

The Java code used for simple regression analysis came from the Apache Commons Mathematics library, statistics ( http://commons.apache.org). That for launching the user’s web browser was from Dem Pilifian ( http://www.centerkey.com/java/browser), modified to run under Java 1.4.

## Results

**Figure 1:**
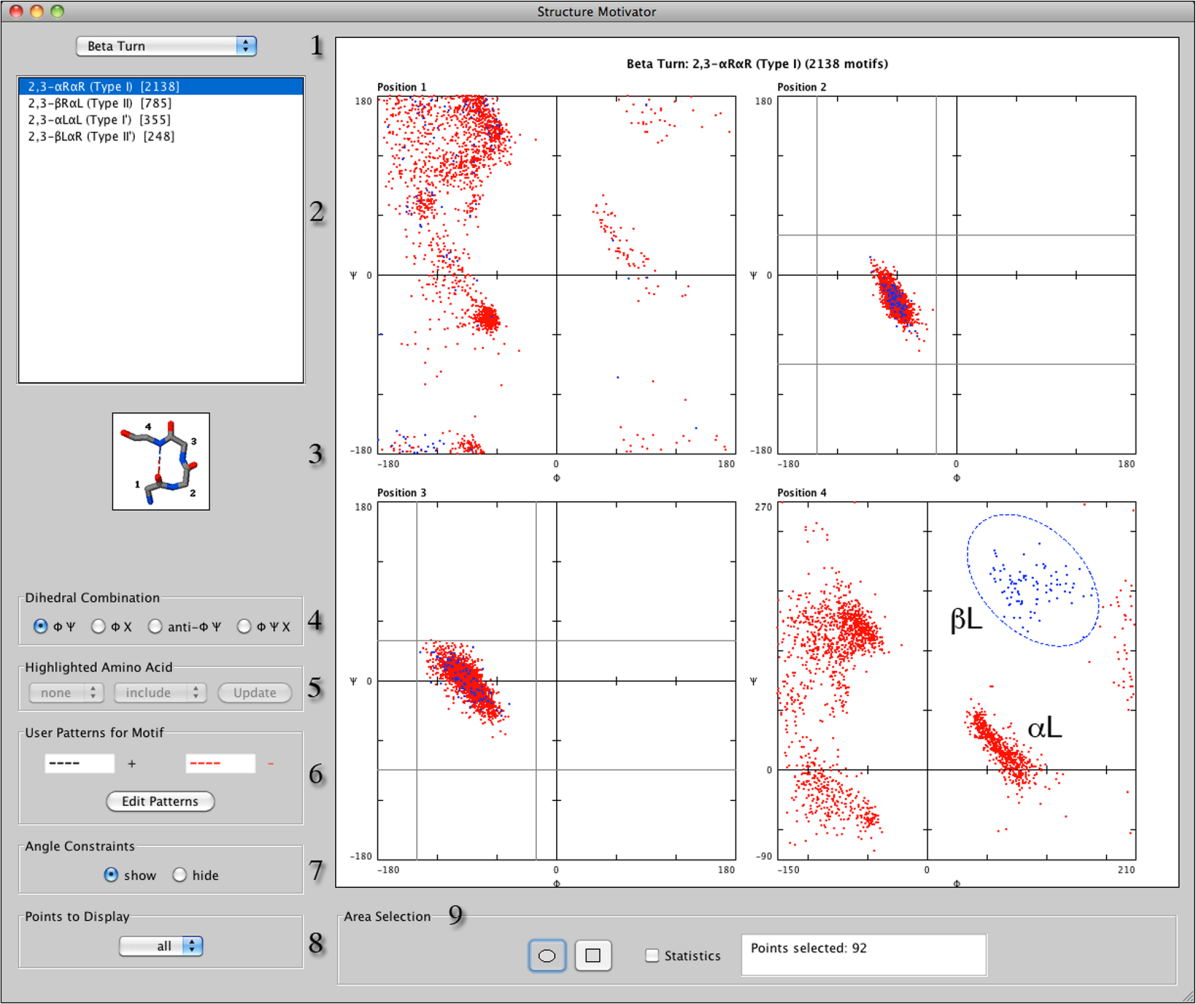
**The Structure Motivator console and interactive selection.** The console of Structure Motivator is shown displaying φψ plots for the residues in one of the motifs from the embedded database (a type I or 2,3-αRαR β-turn). The elliptical selection tool has been used to encompass the βL region of position 4 after adjusting the *y* axis (ψ) to allow this. Points representing the conformation of all the residues in instances of β-turns corresponding to the selection have become highlighted in blue. The grey horizontal and vertical lines in positions 2 and 3 show the user the dihedral angle ranges used to define the embedded motif, and the cartoon indicates its hydrogen-bonding pattern. The grey lines may be switched off using the ‘Angle Constraints’ control on the console.

The functions of Structure Motivator will be presented, together with examples of their use. The console displayed upon starting the application is shown in Figure 1, with some of its components numbered for reference below.

### Loading structural elements

On launching Structure Motivator the user is presented with a display of the first of the inbuilt motifs (derived from the Protein Motif database underlying the Motivated Proteins web facility [5]). The drop-down menu (Figure 1: 1) allows one to choose from the 19 classes of motif, after which one may select a sub-class (there are almost 100 in all) from the list which loads in the window below (Figure 1: 2). For these inbuilt motifs, a cartoon of the structure is displayed (Figure 1: 3).

A second type of structural element may be loaded from within the application through a menu item, ‘Load n-mers’. This gives access to a complete set (90,000) of small peptides (there is a choice of 3-mers to 6-mers) from the proteins in the database, providing a blank canvas, as it were, from which one can define and prepare subsets of structural motifs.

Alternatively text files in Structure Motivator format specifying sets of structural elements can be loaded using a menu item (‘Open File’). This is of particular interest to users who are able to generate their own data for analysis. Files in this format may also be generated by an associated utility, PreMotivator, which contains the same embedded database as Structure Motivator. PreMotivator allows specification of main-chain dihedral angles at different positions in a query peptide of up to nine residues in length (the maximum for Structure Motivator). The application website also contains files for some motifs not present in the Protein Motif database, e.g. γ-turns [20] and catgrips [21], together with some other structural elements, including α-turns, 3_10_ helices, and sections of α-helices.

### Choice of dihedral angles to display

After loading a set of structural motifs or elements, one is presented with separate φψ plots for each residue — the standard Ramachandran plot (Figure 1). The ‘Dihedral Combination’ controls (Figure 1: 4) allow one to change to the alternatives of χ1 angles plotted against φ (φχ1 plot), a linked composite of the φχ1 plot with the φψ plot (φψχ plot), or the anti-φψ plot.

**Figure 2:**
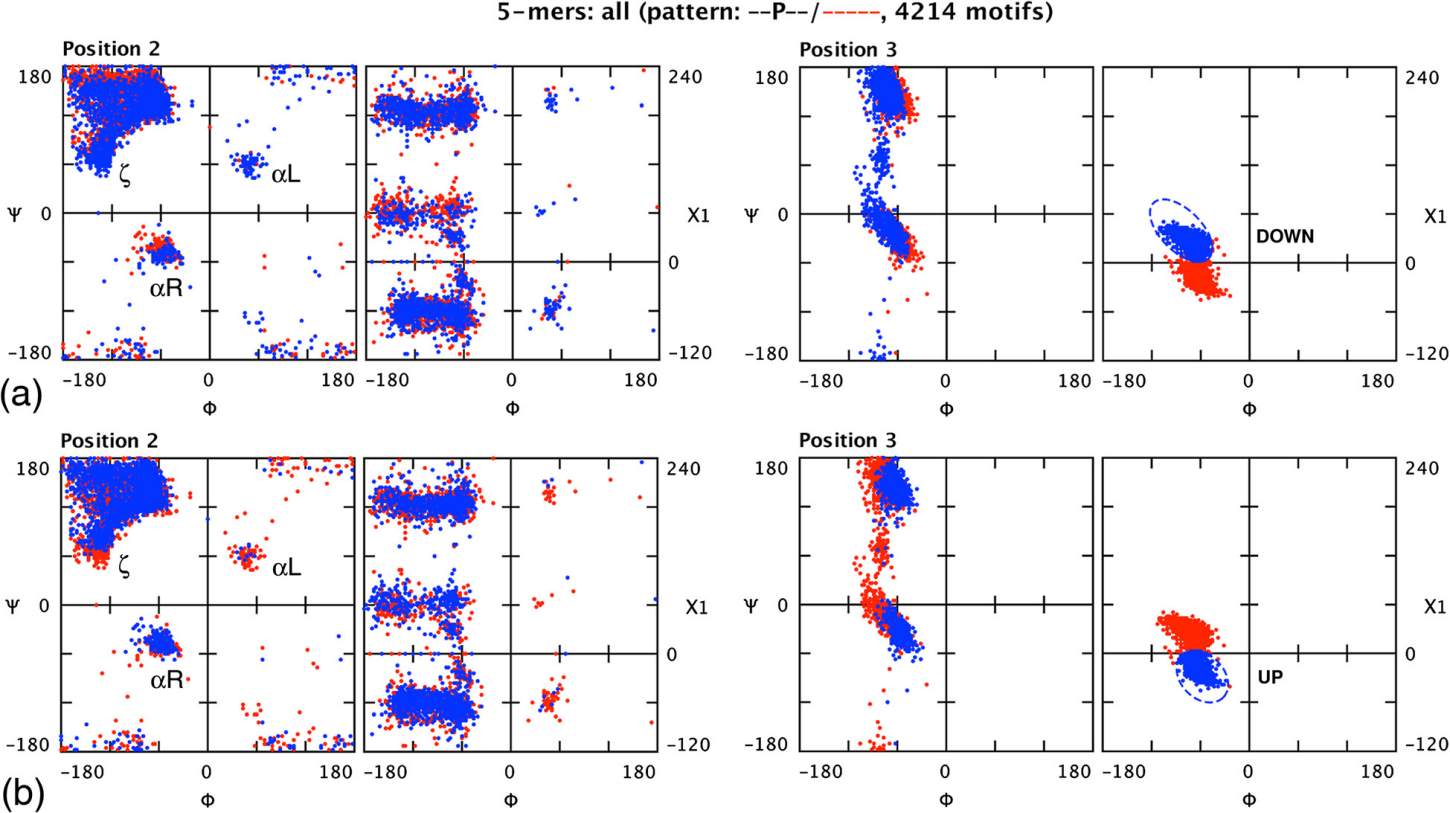
**Inter-relationship between φψ and φχ1 plots.** A set of pentapeptides with pro at position 3 were displayed using the ‘φψχ’ combination setting (Figure 1) and selections made in the χ1 region of position 3 corresponding to (**a**) the DOWN pucker, and (**b**) the UP pucker of the pro ring. Only the plots for positions 2 and 3 are shown. Note that the *y* axis (χ) of the φχ1 portion of the φψχ plot defaults to the range –120˚ to 240˚ to prevent the trans rotamer being split across the 180˚/–180˚ boundary.

The φψχ plot is particularly useful when one is concerned with the inter-relationship between χ1 angles and both φ and ψ angles. An example is provided by pentapeptides with the simple - -P- - sequence motif. Figure 2 shows two alternative χ1 marquee selections at position 3 (made as described below), allowing separate visualization of elements with either the DOWN (Figure 2 (a)) or UP (Figure 2 (b)) pucker of the proline ring. The φψχ plot allows one to see clearly the influence of these different χ1 distributions on the ψ distribution at position 2 — the preceding residue. (This has been observed previously [22,23] and is particularly evident for the ζ [10] and αR regions of the plot. In addition it can be seen how the αL conformation is disfavoured at the position preceding a proline ring with UP pucker.) 

In the anti-φψ plot (see Additional file 2), the ψ angle for one position in a motif is plotted against the φ angle in the following position, allowing study of the pair of angles flanking the peptide bonds, rather than those flanking the α-carbon residues. Such plots are useful for examining peptide-plane flipping [24].

### Modifying the display

The console has controls to modify the way in which the dihedral angle plots are displayed. The number of points plotted may be decreased (Figure 1: 8), which can be useful if there are very many of them, and an option is available (Figure 1: 7) to hide the angle constraints (grey lines at residue positions 2 and 3 in Figure 1) which indicate the ranges of dihedral angles used in the definition of some of the inbuilt motifs.

The two-dimensional nature of a standard Ramachandran plot does not express the 360˚ continuity of dihedral angles, so that a cluster of structural elements may appear both at the top and bottom of a plot, or at its left and right extremities. To facilitate selection of such clusters the user can adjust the axes of the plot by double-clicking at a residue position and entering values in a dialogue box. Such an adjustment, to group together points representing the βL conformation, is illustrated in Figure 1 for residue 4.

Another option (Figure 1: 5) allows one to visualize the distribution of any particular amino acid within the residues of a structural element. One can highlight an amino acid (‘include’ it), ‘exclude’ it to view only the dihedral angle distribution of the other amino acids, or restrict the display to this ‘sole’ amino acid. One use of this facility is to determine whether or not a particular amino acid is evenly distributed within a region of the plot. For example, applying this for glycine in Figure 1 demonstrates its asymmetric distribution in the αL region at residue 4 (see Additional file 3).

### Making selections from the dihedral angle plots

There are two criteria on which selection of a sub-set of structural motifs may be made: amino-acid sequence pattern and dihedral angle distribution at a particular position.

The ‘Edit Patterns’ button on the console (Figure 1: 6) provides access to a dialogue box in which the user may specify a sequence pattern of amino acid residues to be present in a structural motif (displayed in black) and/or a pattern of residues to be excluded (displayed in red). The 4214 instances of the element illustrated in Figure 2 were selected in this way from 90,000 4-mers by specifying the pattern - -P- -. A facility that may be used to inform such sequence-based selection is a pop-up menu of amino-acid composition at any residue position, evoked by a right mouse click in the plot for the residue in question (see Additional file 4).

To select structural elements with a particular range of dihedral angles one chooses either the rectangular or elliptical marquee tool in the ‘Area Selection’ region area of the console (Figure 1: 9) and drags over the area to be selected. The points corresponding to selected instances of the elements are highlighted in blue — both within the dotted outline of the selection marquee and in the plots for the other residues — whereas unselected points remain red (Figure 1). Sometimes it is more convenient to exclude an area of the plot. This can be done by holding down a modifier key when dragging, in which case instances outside the area enclosed by the marquee are selected. As an aid to precise selection one can display the co-ordinates at any point in a ‘tool-tip’ if the cursor is kept stationary at that point for a few seconds (see Additional file 4).

The power of such interactive selection is in defining a subset of structural elements for export. (In Figure 1 one could proceed to export all Type I β-turns with the βL conformation in position 4.) However the tool can also be used analytically. An option in the ‘Area Selection’ region of the console allows display of various statistics: mean values of the angles within the area and the slope of the line through it (see Additional file 4).

### Comparing dihedral angles within elements

**Figure 3:**
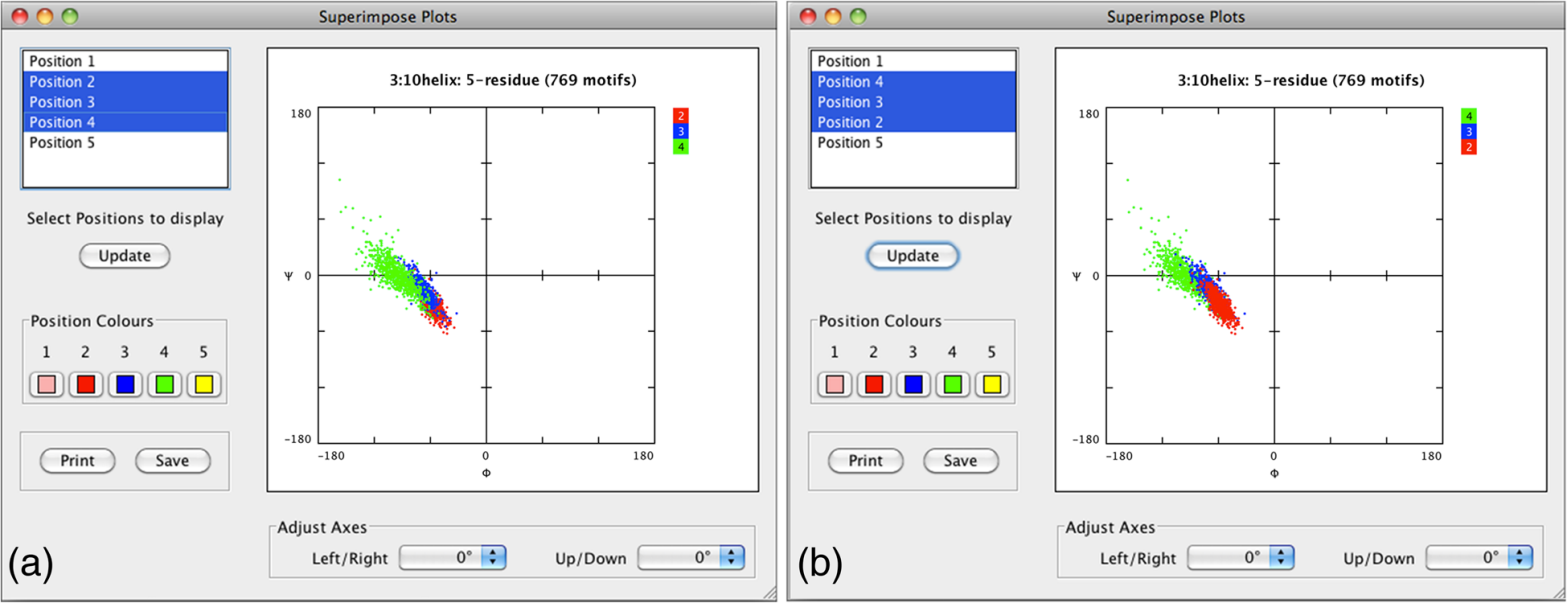
**Superimposition of plots from different positions in a motif.** (**a**) The plot superimposition window of Structure Motivator is shown displaying the φψ distribution for the three central residues of the five-residue 3_10_ helix in a pentapeptide. The pentapeptide includes the *N*-cap (position 1) and the *C*-cap (position 5), the display of which have been suppressed by deselection. Residues are drawn in the order 2 (red), 3 (blue) and 4 (green), so that only the distribution for residue 4 is seen in its entirety. (**b**) As in the foregoing, but with the order reversed so that position 2 is drawn last and its distribution seen in its entirety. The file used for this figure (‘threeTen5.txt’) is included in the package distributed with the application.

Structure motivator provides a facility that allows the distribution of φ/ψ angles at different positions in a structural element to be compared by superimposition (the ‘Superimpose φ/ψ plots’ menu item). An analytical example of the use of this is shown in Figure 3 for the three central residues of five-residue 3_10_ helices (structural elements in an external file derived from SQL queries on our Protein Motif database). It can be seen how the distribution of dihedral angles changes between positions, as has been documented previously [25]. 

Figure 3 also illustrates that irrelevant positions (positions 1 and 5 in this case) may be excluded and that the order of imposition may be altered (cf. Figure 3 (a) and (b)). One can use this facility to prepare figures for publication (e.g. Figure four and Figure five of [26]) as colours may be modified, if necessary, for the output medium, and superimpositions saved or printed.

### Viewing elements in the context of a protein

**Figure 4:**
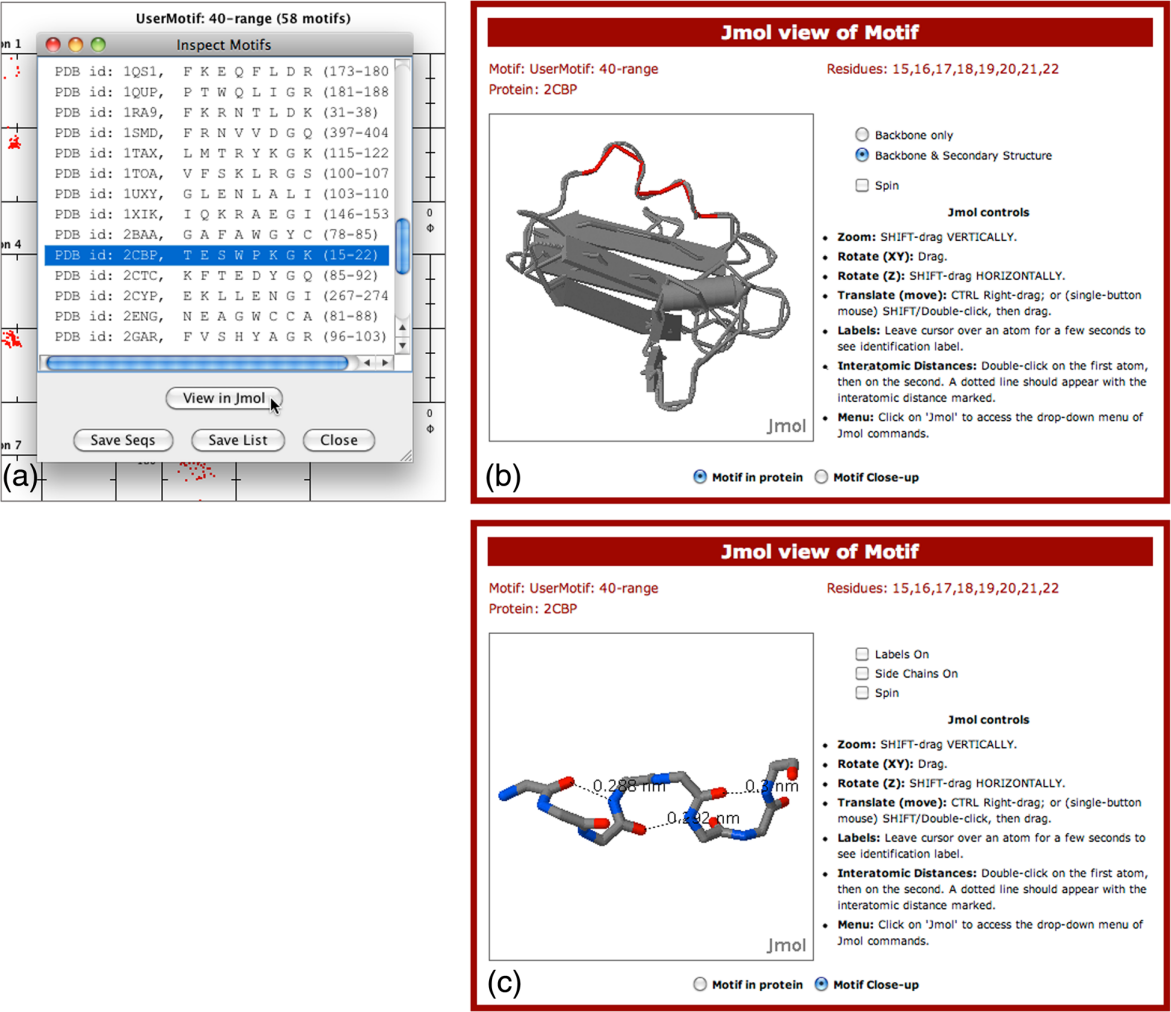
**Inspection of structural elements in the Jmol viewer.** (**a**) The ‘Inspect Motifs’ window of Structure Motivator with the 58 octapeptides described in the text loaded and one selected ready for viewing. (**b**) Selected structural element (2CBP, residues 15–22 [27]), highlighted in red, in secondary structure view in the context of the whole protein. (**c**) Close-up view of the octapeptide with hydrogen bonds visualized. The display of side-chains and residue numbers has been suppressed. The file used to generate the octapeptide in PreMotivator (‘8mer.txt’) is included in the package distributed with the application. The description ‘40-range’ indicates that a range of ± 40˚ for the angles specified in 8mer.txt was used to define the peptide.

A virtue of the dihedral angle plot is that it allows multiple instances of a structural type to be examined. However at a certain stage in analysis one often wishes to examine individual instances in the context of the three-dimensional structure of the proteins in which they reside. The menu option, ‘Inspect Motifs’, lists the motifs in a marquee selection (Figure 4 (a)), and allows the user to select one and view it on a web page (Figure 4 (b)) using the Jmol structure viewer [19]. There are two alternative views of the page — ‘in protein’ (Figure 4 (b)) or ‘close-up’ (Figure 4 (c)) — each with custom controls in addition to Jmol’s own controls. 

The way that this facility might be used is illustrated for a set of 58 octapeptides (generated with PreMotivator) in which the dihedral angles specified at positions 2–7 are those found in three successive β-turns, the first two of type I (2,3-αRαR) and the third of type II (2,3-βRαL). The question that we wished to answer was which, if any, of these elements were *not* parts of α-helices. Using the ‘Inspect Motifs’ facility we loaded each successively into Jmol, turned on the secondary-structure display option, located the octapeptide (highlighted in red), and noted if it fell outside the helices. We processed the 58 structural elements in just ten minutes, identifying six of interest, one of which is shown in Figure 4.

The utility of the close-up view is illustrated in Figure 4 (c). Specifying dihedral angles found in β-turns — as was done in generating the octapeptides — does not in itself guarantee that corresponding hydrogen bonds are present. However using the close-up view one can ‘click-join’ potential hydrogen-bonded atoms and see the length of the putative bond displayed.

### Exporting data

Structure Motivator allows export of different types of data. Pertinent to the objective of sub-categorizing structural elements is export of selections of the type shown in Figure 1 or 2 as files in Structure Motivator format. Such exported text files can be reloaded into Structure Motivator for further analysis or sub-categorization. There are also options to save simpler listings of the primary structures of elements in a selection, either with information identifying their position in a protein (for use when inspecting the motifs in a molecular viewer other than Jmol) or as plain alphabetical strings suitable for computational analysis.

### Other features

The colours with which points are displayed in Structure Motivator can be changed from the Preferences menu item to provide altered contrast for those with impaired colour vision or for the requirements of publication. Facilities for printing and saving graphical visualizations are available from the File menu. Instructions are available from the Help menu within the application, together with links to an on-line glossary of the inbuilt motifs. A manual containing more detailed instructions and information in PDF format (Additional file 5) is distributed with the application and is available on-line.

## Discussion

Identification and analysis of small regions of protein structure has focussed primarily on linear patterns of amino acids, for example those in the Pfam [28] and Prosite [29] databases. Fewer applications are directed specifically at the three-dimensional conformations of such structures, although the DALI tool has been used for this purpose [30] and Ramachandran Plot Explorer allows one to investigate the effects (e.g. on hydrogen bonds) of altering the dihedral angles [31]. The Ramachandran plot is frequently used for visualization or analysis in protein studies, often in relation to a single protein. For example the PROCHECK suite of programs uses Ramachandran plots to check the stereochemical quality of protein structures [32]. However this use is quite different from that in Structure Motivator, and we are not aware of comparable software for the purpose of analysing small structural elements.

The facilities most related to Structure Motivator are in a web application, PDBeMotif (formerly MSDmotif [6]), rather than in a standalone program. PDBeMotif ( http://www.ebi.ac.uk/pdbe-site/pdbemotif/) has comprehensive form-based querying of the whole Protein Data Bank, and presents summary data for many of the motifs from Motivated Proteins. It also provides φ/ψ (but not χ1) plots of each motif, but these are intended for visualization, and interaction (clicking within a plot) is solely to link to the corresponding proteins (cf. our ‘Inspect Motifs’ facility). With Structure Motivator, in contrast, interaction using the marquee tool allows sub-sets within a broad group to be selected for further analysis, and we regard this as the distinguishing feature of the application.

An embedded relational database of 429 high-resolution protein structures [5] underpins Structure Motivator. This was ported from MySQL to Derby, a different database management system written in Java and designed to allow databases to be incorporated into programs. The database allows the program to generate peptide ‘templates’ from which users can prepare their own structural elements, either within the application itself, or with the auxiliary tool, PreMotivator.

Although we have provided a tool for use by structural biologists without database or programming skills, we recognize that it is not without limitations. The embedded database within Structure Motivator is restricted to 429 proteins, albeit high-resolution structures with added fixed hydrogen atoms and corrected to ensure optimal orientations of asn and gln [33]. Nevertheless, if users wish to examine structural elements from proteins not represented in this set they need to derive them elsewhere and import them into Structure Motivator. The other limitation is that we do not provide a tool for users to prepare structural motifs with particular specified hydrogen-bonding patterns, in part because SQL queries involving hydrogen bonds can be very slow to run. The motifs provided do present several hydrogen-bonding patterns that may be useful as starting points, and we have shown how the customized Jmol view provided allows sub-classes of structural elements to be examined for hydrogen bonds (Figure 4 (c)).

## Conclusions

We have demonstrated how Structure Motivator can be employed as a research tool to analyse and sub-classify either the inbuilt motifs provided or a user’s own set of external structural elements. Its repertoire of tools can be used to analyse any peptide with a definable structure: all that is necessary is that the peptide have a fixed number of residues and a common reference point. As an example, we have used Structure Motivator to analyse hexapeptides in which the third residue is the *C*-terminus of an α-helix [26] — not what one might normally regard as a ‘motif’— and then sub-divided these hexapeptides by making selections based on the conformation at the *C*-cap (residue 4). Other structural elements that we have analysed in our published research are α-turns and six-residue 3_10_ helices [26], and examples from our unpublished work include β-hairpins, αRαL repeats, and peptides containing residues with dihedral angles in the ζ-region of the Ramachandran plot.

Structure Motivator provides functionality not found in other applications for investigating protein structure. Equally important are the ease, speed and immediacy with which this functionality can be employed. Consider, for example, the ζ -region of the dihedral-angle plot in Figure 2, and how much easier, quicker and more accurate it is to select this with an elliptical marquee tool than by making the corresponding SQL query. The visualizations available for the structural subsets in the resulting selections themselves suggest new queries, which can be rapidly made by a succession of further selections. Thus, Structure Motivator is a unique “What if?” tool for investigating the three-dimensional structure of proteins: it both provokes ideas for experimental avenues and provides the means by which one may explore them.

## Availability and requirements

Project name

Structure Motivator

Project home page

http://motif.gla.ac.uk/motivator.html

Operating system(s)

Platform independent

Programming language

Java

Other requirements

Java 1.4 or higher. Internet connection and web browser with Java support for inspecting individual structures using the Jmol applet.

License

GPL

Restrictions to non-academic use

License required

## Abbreviations

αL: A residue conformation represented by φ values between 20˚ and 140˚, and ψ values between –40˚ and 90˚.; αR: A residue conformation represented by φ values between –140˚ and –20˚, and ψ values between –90˚ and 40˚.; βL: A residue conformation represented by φ values between 20˚ and 160˚, and ψ values between –180˚ and –80˚.; βR: A residue conformation represented by φ values between –160˚ and –20˚, and ψ values between 80˚ and 180˚.; SQL: Structured Query Language.

## Competing interests

The authors declare that they have no competing interests.

## Authors’ contributions

The original idea for a desktop application with an embedded database was that of EJMW, and he tested prototypes and provided feedback during development. DPL was responsible for software design and implementation, and originated many of the features for using the application as an investigative tool. Both authors contributed to the writing of the manuscript, with DPL making the initial draft. Both authors read and approved the final manuscript.

## Supplementary Material

Additional file 1 ***Schema of embedded database.****The file shows those parts of the Protein Motif Database*[5]*retained in the embedded database in Structure Motivator.* Click here for file

Additional file 2 ***Statistical information and the anti-φψ plot.****The file shows the anti-φψ plot for 2,3,-αRαR (5-residue) β****-****bulge loops. The points representing the combination of the ψ angle at position 2 and the φ angle at position 3 have been selected, and their mean values and the slope of the line through them is displayed in the statistics window of the console.* Click here for file

Additional file 3 ***Visualization of specific amino acids.****The file shows the αL region at position 4 of a φψ plot of the type I β-turn. The three views shown are after console selection of gly as the ‘Highlighted Amino Acid’, with the respective options ‘include’, ‘exclude’ and ‘sole’. The selected amino acid, gly is coloured blue whereas other amino acids are coloured red.* Click here for file

Additional file 4 ***Visualizing amino acid composition and dihedral angles.****The file shows the φψ plot at residue 4 of a type I β-turn. A pop-up listing the amino acid composition at this residue has been evoked by right-clicking within the plot, and a pop-up indicating the co-ordinates at the tip of the arrow cursor has been evoked by keeping it stationary for a few seconds.* Click here for file

Additional file 5 ***Structure Motivator Manual.****The file contains a detailed description of the operation of Structure Motivator and PreMotivator.* Click here for file

## References

[B1] Structural alignment software [ http://en.wikipedia.org/wiki/Structural_alignment_software]

[B2] Orengo CA Michie AD Jones S Jones DT Swindells MB Thornton JM CATH — a hierarchic classification of protein domain structures Structure 1997 5 1093 1108 10.1016/S0969-2126(97)00260-8 9309224

[B3] Hubbard TJP Ailey B Brenner SE Murzin AG Chothia C SCOP: a Structural Classification of Proteins database Nucleic Acids Res 1999 27 254 256 10.1093/nar/27.1.254 9847194 PMC148149

[B4] Venkatachalam CM Stereochemical criteria for polypeptides and proteins. V. Conformation of a system of 3 linked peptide units Biopolymers 1968 6 1425 1436 10.1002/bip.1968.360061006 5685102

[B5] Leader DP Milner-White EJ Motivated proteins: a web application for studying small three-dimensional protein motifs BMC Bioinforma 2009 10 60 10.1186/1471-2105-10-60 PMC2651126 19210785

[B6] Golovin A Henrick K MSDmotif: exploring protein sites and motifs BMC Bioinforma 2008 9 312 10.1186/1471-2105-9-312 PMC2491636 18637174

[B7] Ramachandran GN Ramakrishnan C Sasisekharan V Stereochemistry of polypeptide chain configurations J Mol Biol 1963 7 95 99 10.1016/S0022-2836(63)80023-6 13990617

[B8] Swindells MB MacArthur MW Thornton JM Intrinsic ϕ, ψ propensities of amino acids, derived from the coil regions of known structures Nat Struct Biol 1995 2 596 603 10.1038/nsb0795-596 7664128

[B9] Kleywegt GJ Jones TA Phi/psi-chology: Ramachandran revisited Structure 1996 4 1395 1400 10.1016/S0969-2126(96)00147-5 8994966

[B10] Karplus PA Experimentally observed conformation-dependent geometry and hidden strain in proteins Protein Sci 1996 5 1406 1420 10.1002/pro.5560050719 8819173 PMC2143451

[B11] Walther D Cohen FE Conformational attractors on the Ramachandran map Acta Crystallogr D 1999 55 506 517 10.1107/S0907444998013353 10089363

[B12] Hovmöller S Zhou T Ohlson T Conformations of amino acids in proteins Acta Crystallogr D 2002 58 768 776 10.1107/S0907444902003359 11976487

[B13] Lovell SC Davis IW Arendall WB de Bakker PIW Word JM Prisant MG Richardson JS Richardson DC Structure validation by Cα geometry: ϕ, ψ and Cβ deviation. Proteins 2003 50 437 450 10.1002/prot.10286 12557186

[B14] Ho BK Thomas A Brasseur R Revisiting the Ramachandran plot: hard-sphere repulsion, electrostatics, and H-bonding in the alpha-helix. Protein Sci 2003 12 2508 2522 14573863 10.1110/ps.03235203 PMC2366959

[B15] Betancourt MR Skolnick J Local propensities and statistical potentials of backbone dihedral angles in proteins. J Mol Biol 2004 342 635 649 10.1016/j.jmb.2004.06.091 15327961

[B16] Pavelcik F Vanco J Simple procedure for conformation-family search in multidimensional torsion-angle space J Appl Cryst 2006 39 315 319 10.1107/S0021889806005589

[B17] DdlUtils [ http://db.apache.org/ddlutils/]

[B18] Apache Derby [ http://db.apache.org/derby/]

[B19] Herráez A Biomolecules in the computer: Jmol to the rescue Biochem Mol Biol Educ 2006 34 255 261 10.1002/bmb.2006.494034042644 21638687

[B20] Milner-White EJ Situations of gamma-turns in proteins. Their relation to alpha-helices, beta-sheets and ligand binding sites J Mol Biol 1990 216 385 397 10.1016/S0022-2836(05)80329-8 2254936

[B21] Watson JD Milner-White EJ The conformations of polypeptide chains where the main-chain parts of successive residues are enantiomeric. Their occurrence in cation and anion-binding regions of proteins J Mol Biol 2002 315 183 191 10.1006/jmbi.2001.5228 11779238

[B22] Ho BK Brasseur R The Ramachandran plots of glycine and pre-proline BMC Struct Biol 2005 5 14 10.1186/1472-6807-5-14 16105172 PMC1201153

[B23] Ho BK Coutsias EA Seok C Dill KA The flexibility in the proline ring couples to the protein backbone Protein Sci 2005 14 1011 1018 10.1110/ps.041156905 15772308 PMC2253451

[B24] Hayward S Peptide-plane flipping in proteins Protein Sci 2001 10 2219 2227 11604529 10.1110/ps.23101 PMC2374056

[B25] Enkhbayar P Hikichi K Osaki M Kretsinger RH Matsushima N 3(10)-helices in proteins are parahelices. Proteins 2006 64 691 699 10.1002/prot.21026 16783793

[B26] Leader DP Milner-White EJ The structure of the ends of α-helices in globular proteins: Effect of additional hydrogen bonds and implications for helix formation Proteins 2011 79 1010 1019 10.1002/prot.22942 21287629

[B27] Guss JM Merritt EA Phizackerley RP Freeman HC The structure of a phytocyanin, the basic blue protein from cucumber, refined at 1.8Å resolution. J Mol Biol 1996 262 686 705 10.1006/jmbi.1996.0545 8876647

[B28] Punta M Coggill PC Eberhardt RY Mistry J Tate J Boursnell C Pang N Forslund K Ceric G Clements J The Pfam protein families database Nucleic Acids Res 2012 40 D290 301 10.1093/nar/gkr1065 22127870 PMC3245129

[B29] Sigrist CJA Cerutti L Hulo N Gattiker A Falquet L Pagni M Bairoch A Bucher P PROSITE: a documented database using patterns and profiles as motif descriptors Brief Bioinformatics 2002 3 265 274 10.1093/bib/3.3.265 12230035

[B30] Holm L Sander C Mapping the protein universe Science 1996 273 595 603 10.1126/science.273.5275.595 8662544

[B31] Ho BK The Ramachandran Plot Explorerhttp://boscoh.com/ramaplot/

[B32] Laskowski RA MacArthur MW Moss DS Thornton JM PROCHECK: a program to check the stereochemical quality of protein structures J Appl Cryst 1993 26 283 291 10.1107/S0021889892009944

[B33] Word JM Lovell SC Richardson JS Richardson DC Asparagine and glutamine: using hydrogen atom contacts in the choice of side-chain amide orientation J Mol Biol 1999 285 1735 1747 10.1006/jmbi.1998.2401 9917408

