# Supplementary figures and images for: Structure Motivator: A tool for exploring small three-dimensional elements in proteins
Source: BMC Struct Biol. 2012 Oct 16;12:26. doi: 10.1186/1472-6807-12-26 (PMC3507813; doi:10.1186/1472-6807-12-26)

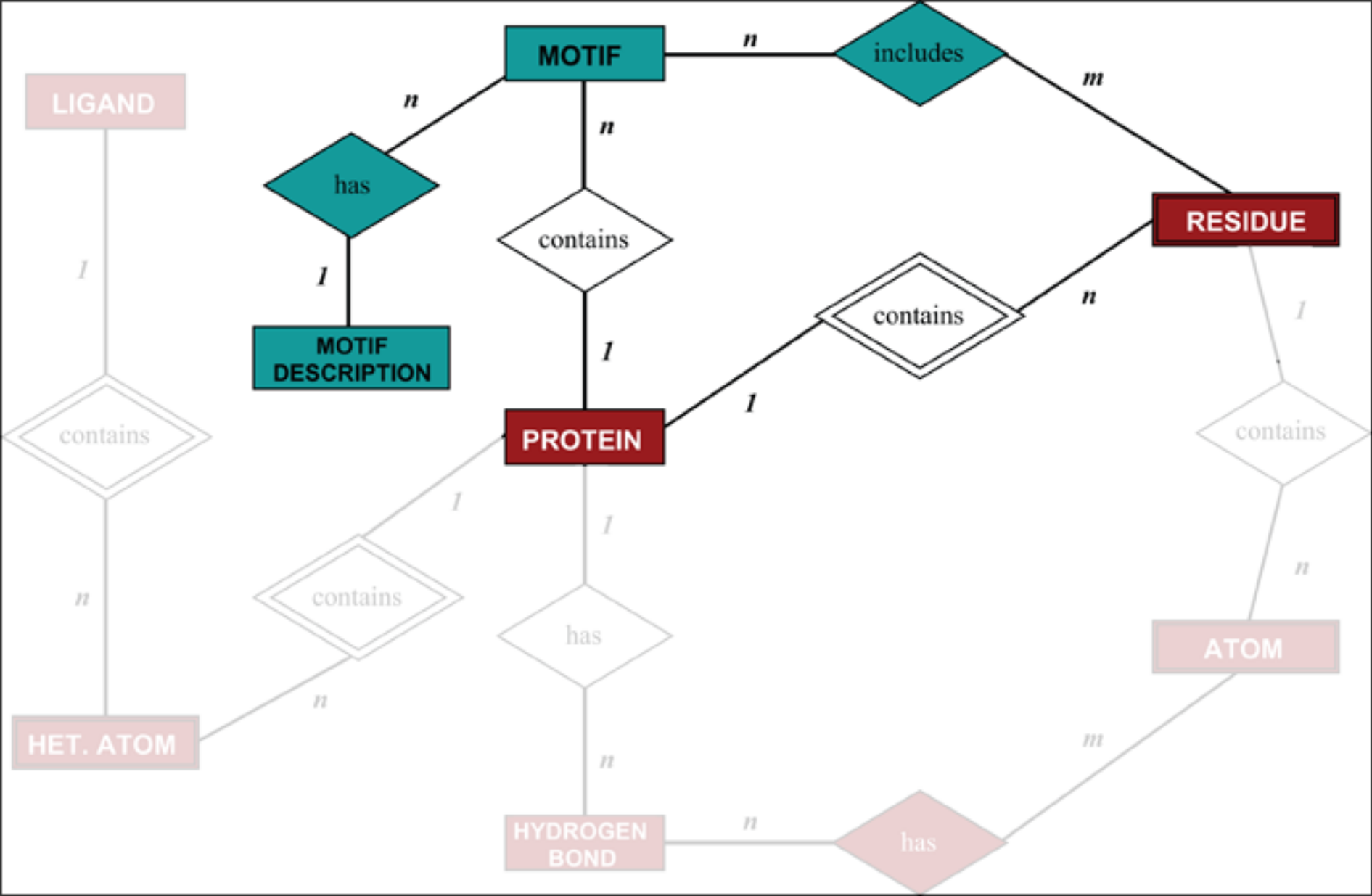

Supplement: Additional file 1 — Schema of embedded database.The file shows those parts of the Protein Motif Database[5]retained in the embedded database in Structure Motivator. [file 1472-6807-12-26-S1.pdf]

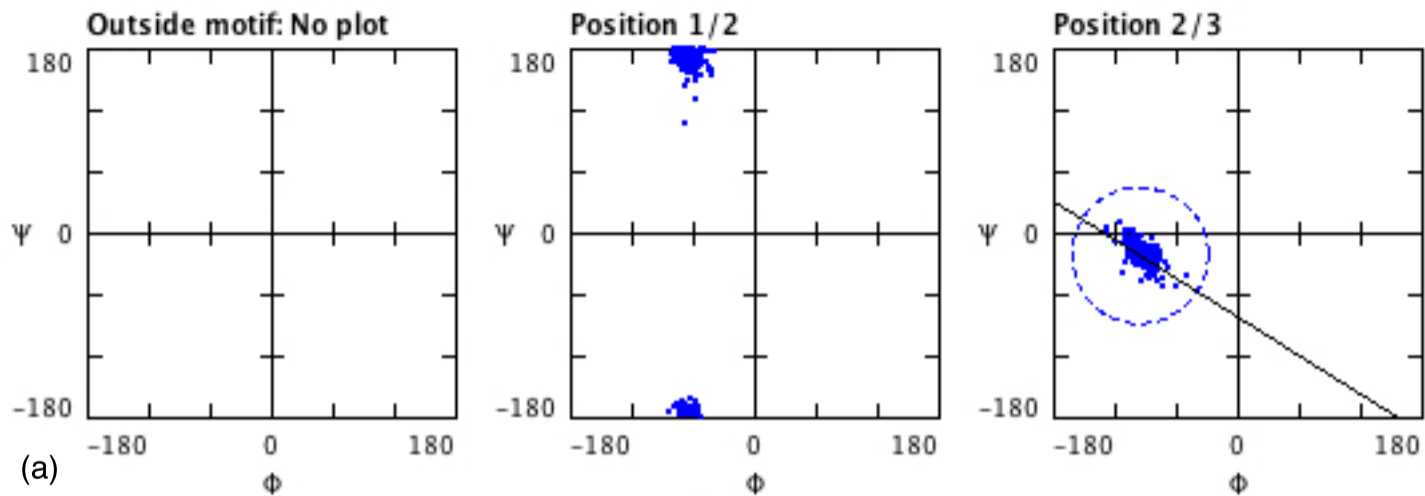

(b)

Slope:  $-32^\circ$  ( $r^2 = 0.41$ )

Means:  $\phi = -90.7^\circ$ ,  $\Psi = -22.6^\circ$

Supplement: Additional file 2 — Statistical information and the anti-φψ plot.The file shows the anti-φψ plot for 2,3,-αRαR (5-residue) β-bulge loops. The points representing the combination of the ψ angle at position 2 and the φ angle at position 3 have been selected, and their mean values and the slope of the line through them is displayed in the statistics window of the console. [file 1472-6807-12-26-S2.pdf]

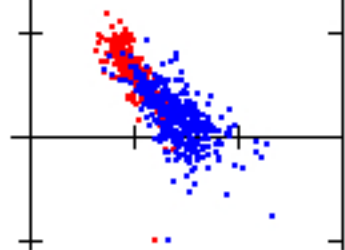

include

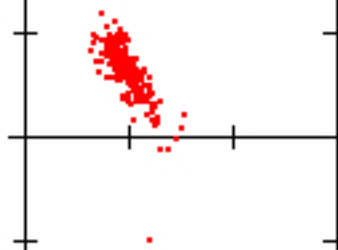

exclude

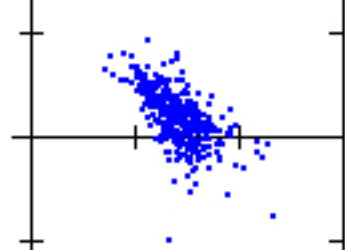

sole

Supplement: Additional file 3 — Visualization of specific amino acids.The file shows the αL region at position 4 of a φψ plot of the type I β-turn. The three views shown are after console selection of gly as the ‘Highlighted Amino Acid’, with the respective options ‘include’, ‘exclude’ and ‘sole’. The selected amino acid, gly is coloured blue whereas other amino acids are coloured red. [file 1472-6807-12-26-S3.pdf]

## Position 4

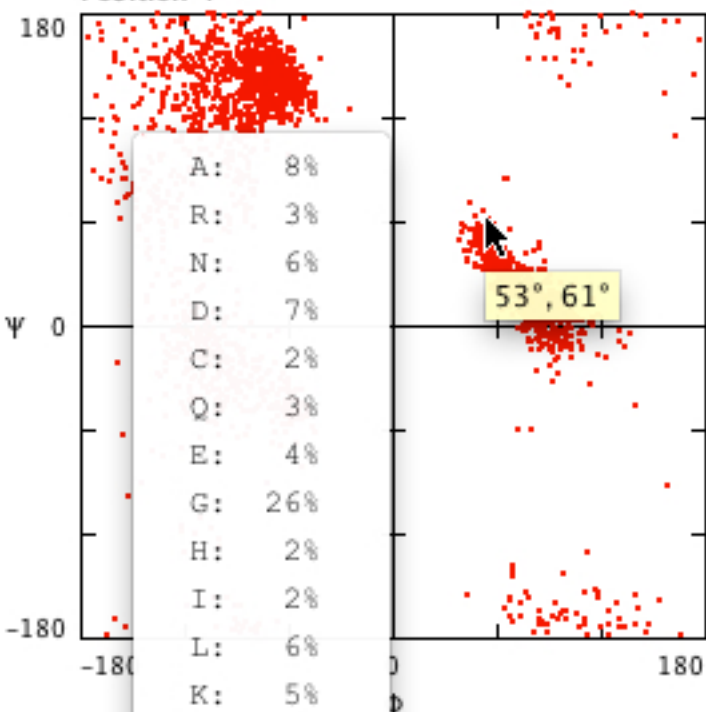

Supplement: Additional file 4 — Visualizing amino acid composition and dihedral angles.The file shows the φψ plot at residue 4 of a type I β-turn. A pop-up listing the amino acid composition at this residue has been evoked by right-clicking within the plot, and a pop-up indicating the co-ordinates at the tip of the arrow cursor has been evoked by keeping it stationary for a few seconds. [file 1472-6807-12-26-S4.pdf]
