# Supplementary material for: Structure Motivator: A tool for exploring small three-dimensional elements in proteins
Source: BMC Struct Biol. 2012 Oct 16;12:26. doi: 10.1186/1472-6807-12-26 (PMC3507813; doi:10.1186/1472-6807-12-26)
Supplement: Additional file 5 — Structure Motivator Manual.The file contains a detailed description of the operation of Structure Motivator and PreMotivator. [file 1472-6807-12-26-S5.pdf]

Structure Motivator

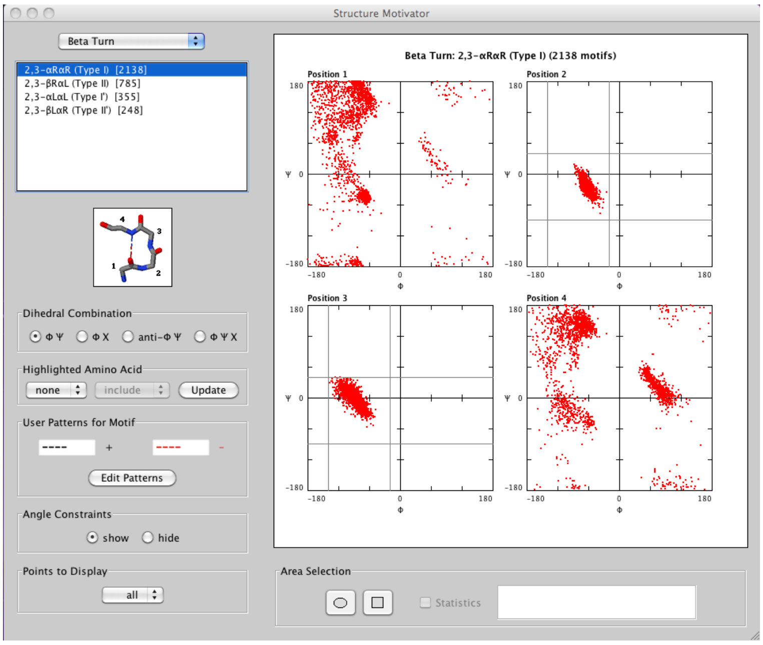

User's Manual

CONTENTS

1. Introduction 3

2. Using *Structure Motivator* 4

3. Menu and Console Reference 11

4. File Format for Import and Export 17

Appendix I: The Database: Proteins and Motifs 18

Appendix II: Statistics 18

Appendix III: *PreMotivator* 19

Structure Motivator v. 2.5.1

© David P. Leader & E. James Milner-White 2009–12

1. Introduction

Structure Motivator (formerly *Motif Dihedralizer*) is a desktop application for studying the range of structures represented by particular small three-dimensional protein motifs. It offers a variety of tools to allow users to manipulate and explore a range of motifs built into the application and an ever-increasing selection available as downloadable text files on the *Motivated Proteins* website. The tools can also be used with the user's own data sets. The key features of the application are:

- The relationship between ϕ, ψ and $\chi 1$ dihedral angles can be examined simultaneously.
- The ϕ and ψ angles at either side of a peptide bond can also be studied.
- Interactive selection of areas of the plots can be made.
- Selection of a subset of motifs can be made on the basis of amino acid sequence.
- Selected motifs can be listed, exported or viewed in a 3D web application.
- Graphs from different positions in a motif may be superimposed on one another.

In addition, there are facilities for import, export, printing, saving graphics in PNG format, statistical analysis, and adjustment of the graph axes for optimal visualization and manipulation. A utility, *PreMotivator*, is availabe for preparing files for import, and is described in an appendix.

System Requirements

Structure Motivator is a cross-platform application, and there are versions for Mac OS X, Windows, and Unix/Linux. Support for Java (version 1.4.2 or higher) is required, which is standard in Mac OS X Tiger and later, and can be downloaded from Oracle's website if necessary for Windows and Unix/Linux. The Mac and Windows versions behave like normal applications for these platforms, require no special installation, and can be run from anywhere in one's filespace provided that directories (folders) do not contain special characters. Instructions are provided in the download for running the Unix/Linux version from the command line. Because an embedded version of the *Protein Motif* database is included in *Structure Motivator*, it only requires an internet connection if one wishes to access the Motif Glossary or to view motifs in three dimensions using the *Jmol* applet.

Acknowledgements

Gary Gray suggested using Apache Derby as an embedded database, and Attila Tajlil ported to Derby the relevant portions of the MySQL database used for *Motivated Proteins*.

Citation in Publications

If you publish work in which you have used *Structure Motivator*, please check whether a paper describing it has appeared. If not, cite:

http://motif.gla.ac.uk/motivator.html

Contacting the Authors

The authors may be contacted via the website above, or directly to david.leader@glasgow.ac.uk or james.milner-white@glasgow.ac.uk. Bug reports, feedback and feature requests are welcomed.

2. Using Structure Motivator

This section contains five scenarios designed to ‘walk’ the user quickly through the main features of *Structure Motivator*. Section 3 provides a systematic reference for menu items and controls. Section 4 describes the import and export file format.

φψ plots and amino-acid visualization

1. Double-click the application icon to launch *Structure Motivator* in Mac OS X or Windows, or follow the instructions provided for launching from the command line on Unix/Linux. When the program opens the alphabetical first motif will be selected and loaded by default.

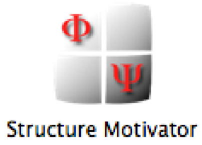

2. Choose ‘Beta Turn’ from the motif selection drop-down category menu. ‘2,3-αRαR (Type I)’ will be selected in the sub-category window, with a cartoon graphic of this structure in the frame below. The main display area contains four φψ (Ramachandran) plots, one for each of the positions in the motif, numbered as in the cartoon. (This is illustrated on the front cover.)

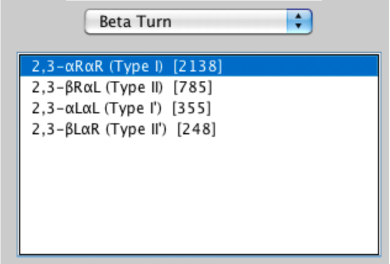

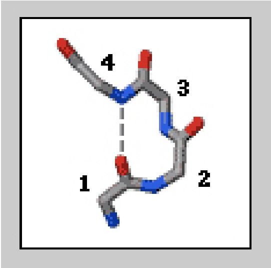

3. The plots for motif positions 2 and 3 have grey horizontal and vertical lines. These represent dihedral angle constraints implicit in the definition of the motif, which is why in these positions all the points fall within the central rectangles produced by the intersection of the grey lines.

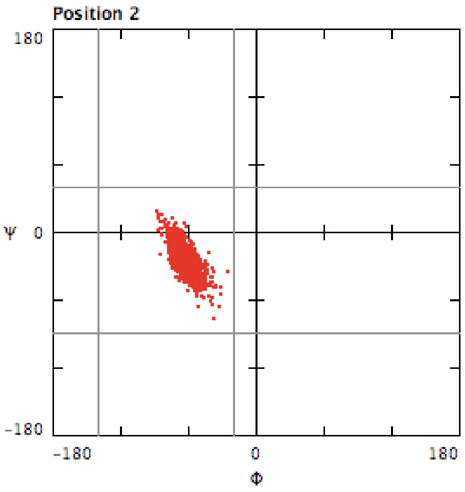

4. Right-click (or control-click if using a Mac one-button mouse) within position 4. A pop-up appears summarizing the amino acid composition at this position. You will see that there is a high proportion of glycine (‘G’) here.

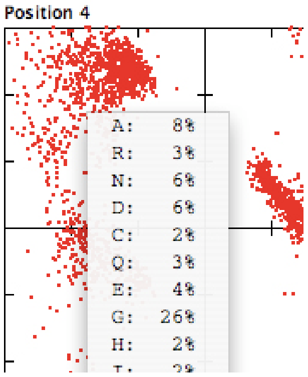

5. In the first drop-down menu of the ‘Highlighted Amino Acid’ section of the Console select ‘gly’. Notice that ‘include’ becomes undimmed in the second drop-down menu. This is the option we require, so click on the ‘Update’ button, which will cause the points for glycine to become coloured blue, in contrast to the other amino acids which are red. (The colours can be changed from the ‘Preferences’ menu.)

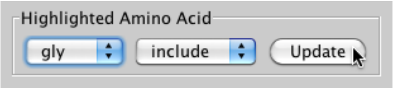

6. It is evident that glycine occupies the ‘south-east’ tail of the αL region of position 4, but because the selected amino acid is drawn last it is unclear whether other amino acids are also present at this position. To address this question select ‘exclude’ in the right-hand drop-down menu, and click the ‘Update’ button. One can see that far fewer of the other amino acids have negative ψ values in the αL cluster. Select ‘sole’ and update the graph to confirm the different distribution.

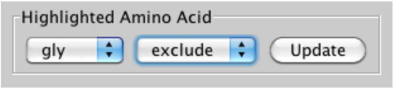

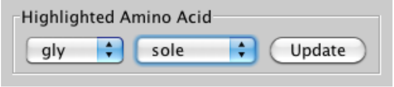

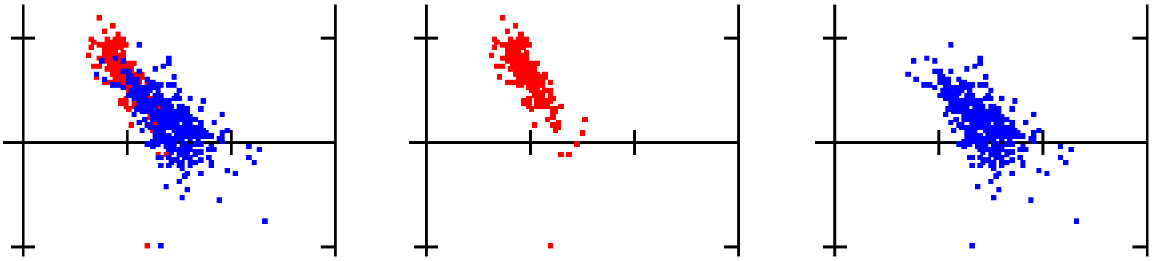

include gly ***exclude gly*** ***sole gly***

7. To prepare the display for printing and saving, first revert to ‘include’ in the ‘Highlighted Amino Acid’ section of the Console. Then click the ‘hide’ button in the ‘Angle Constraints’ section of the Console. The grey lines will disappear.

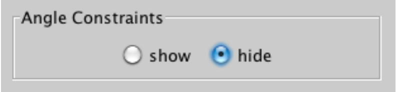

8. We are interested in how the points in the plot cluster, but there are artificial division of the βR and βL clusters in positions 1 and 4. Double-click within the graph for position 1. A dialogue box appears with the option of adjusting the graphs left or right and up or down. Make the selection shown to move the axes down 60° and click ‘OK’. The graph adjusts as shown below. Do likewise for position 4.

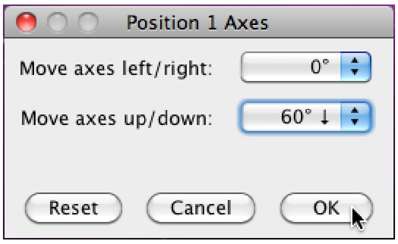

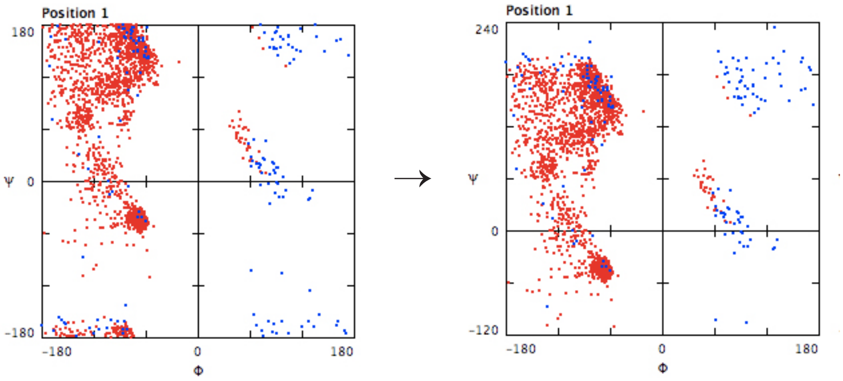

11. Select ‘Print’ from the ‘File’ menu and landscape view in the ‘Page Setup’ dialogue. Print. (If the graphic does not fit on the paper, resize the application window and try again.)
12. Select ‘Save as PNG’ from the ‘File’ menu and save to your filespace. The graphics are suitable for inclusion in a web page, but are not of publication quality.

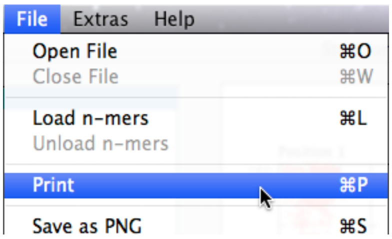

13. If you wish publication-quality graphics, in Mac OS X chose ‘Save as PDF’ in the print dialogue box, or in Windows chose the appropriate option for a third-party PDF generator such as *PDFCreator*. (To ensure that the quality of the PDF file is preserved it may be best to import and edit it in a vector-graphics application such as *Adobe Illustrator*.)

$\phi\chi$ plots, area selection and export

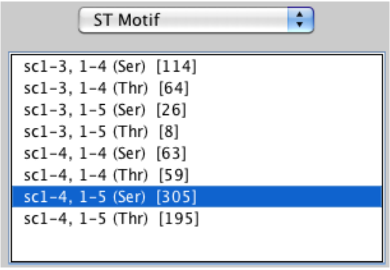

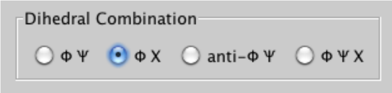

1. For this section we shall start with the ST Motif, in which one of the two hydrogen bonds involves the serine or threonine side-chain OH. Select ‘ST motif’ from the category drop-down menu, ‘sc1-4, 1-5 (Ser)’ in the sub-category window, and then click the ‘Dihedral Combination’ button $\phi\chi$.
2. A section of the resulting display is shown below. The purpose of the $\phi\chi$ option is to visualize the $\chi1$ dihedral angles — the function of the ϕ axis of the plot being to spread the points out so that one can get a visual estimate of relative proportions. In general the $\chi1$ angles of amino acid side-chains tend to fall at approximately –60˚, 60˚ and 180˚. (Proline is exceptional, and accounts for the apparent anomaly in Position 2.)

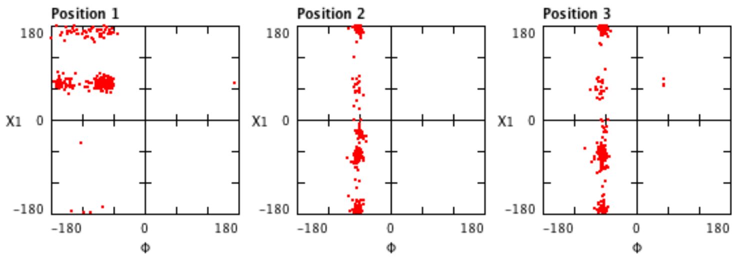

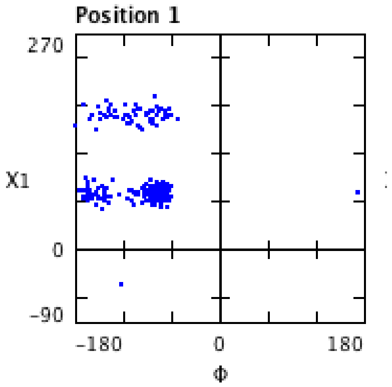

3. Position 1 (serine) of this motif is constrained to $\chi1$ angles of approx. 60˚ and 180˚. Set ‘Highlight Amino Acid’ to include ‘ser’, click the ‘Update’ button, double-click in the graph for position 1, and shift the graph down by 90˚ to see this better.
4. The shift of scale also allows one to select the two groups properly and measure the mean value of $\chi1$. Click on the button for the rectangular selection tool in the ‘Area Selection’ section of the Console. Note that this over-rides the amino acid selection, resetting the colour to red. Now select the upper cluster of points by dragging the cross-hair cursor in the standard manner. You may start to drag outside the graph, but must finish within it. The points within the selection ‘marquee’ are blue, and the display box shows their number (here 62).
5. Click on the ‘Statistics’ button to select it. A horizontal line is drawn through the points, and the mean value appears in the display box. (The line is forced to horizontal only for the $\phi\chi$ option — in other cases the best line through the points is drawn and the angle given, as described in the next section.)

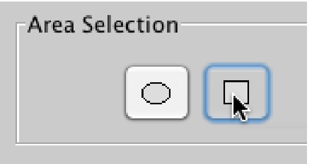

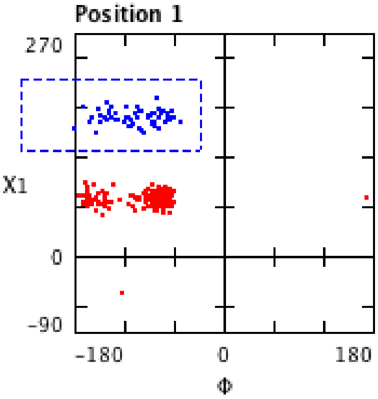

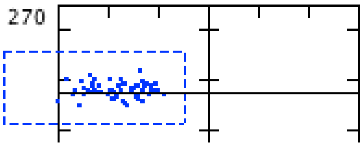

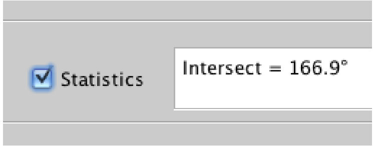

6. The question arises as to whether there is any relationship between these two groups of $\chi1$ angles at residue 1 of the motif and the values of the $\phi\psi$ angles. This is best addressed by switching the ‘Dihedral Combination’ to $\phi\psi\chi$ mode.

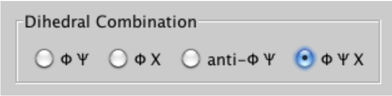

7. Select the upper χ1 region in position 1. It can now be seen that this corresponds to one of two φψ regions at that position.

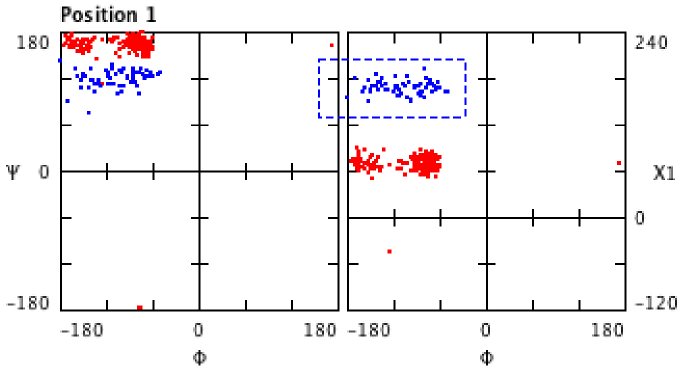

8. One might well wish to divide this motif sub-category on the basis of the χ1 angle in order to facilitate further study. With the marquee selection still in place, choose ‘Save Motif Set from Area Selection’ in the File menu, and save with a suitable name. The output file is plain text with the extension ‘.txt’, the format of which is described in Section 4. The format of the saved text file is suitable for re-import without further editing, although you might wish to edit the sub-category line of the header.

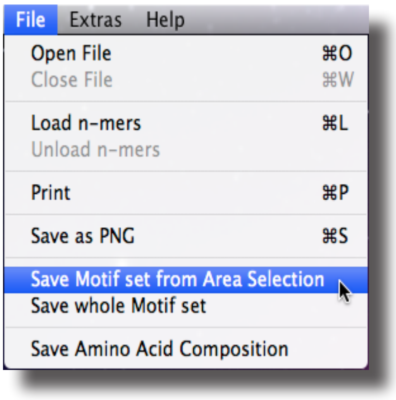

The anti-Ramachandran plot

1. ‘Anti-Ramachandran’ is a term for a plot of dihedral angles in which the ψ angle of one residue is plotted against the φ angle of the *following* residue. The significance of this pair of angles is that they define the conformation of the plane of the peptide bond between the two residues, which is important in certain motifs.
2. Select ‘Beta Bulge Loop’ from the category drop-down menu, accept the default ‘2,3,-αRαR (5-residue)’ sub-category, and click the anti-φψ button in the ‘Dihedral Combination’ section. The nature of this type of plot means that there is one graph fewer than the number of positions in a motif.
3. An aspect of interest in the anti-Ramachandran plot of certain peptide bonds is whether the points have the diagonal distribution often found at the αR and αL positions of a standard φψ plot. Click on either of the ‘Area Selection’ tool buttons, select the cluster of points at position 2/3, and click the ‘Statistics’ button. A diagonal line is drawn, and the mean values of φ and ψ are presented in the statistics window.

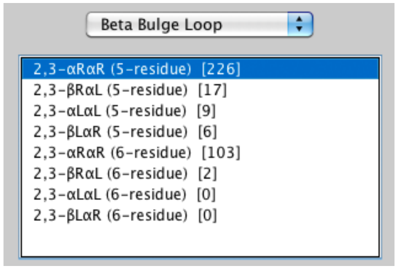

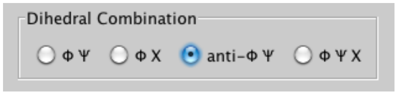

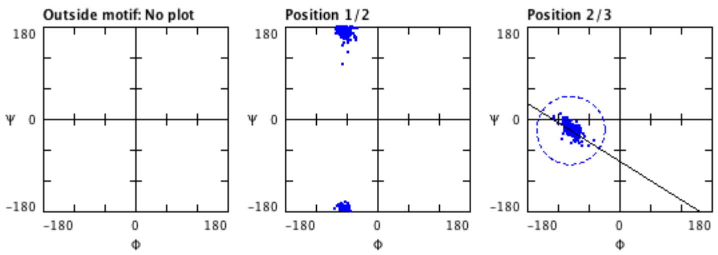

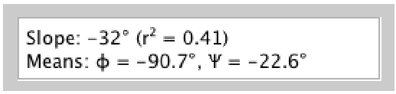

Imposing patterns and inspecting motifs

1. A basic feature of *Structure Motivator* is the ability to select a subset from a group of motifs. As well as selecting a range of dihedral angles using the mouse, one can specify a pattern of amino acids to which motifs must conform. We shall illustrate this with the abundant 1,2-αRαL sub-category of the 'Nest' motif. The φψ plots for this motif are shown below.

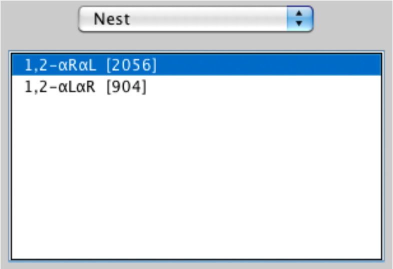

Nest: 1,2-αRαL (2056 motifs)

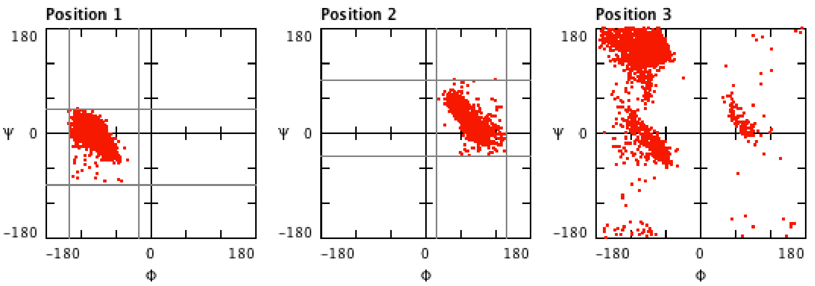

2. Inspection of the amino acid compositions (by right-clicking in the plots) indicates that glycine predominates at position 2, and threonine is quite well represented at position 1. Let us suppose we wish to examine that subset of the Nest motifs that has threonine at position 1 but lacks glycine at position 2. Click the 'Edit Patterns' button in the 'User Patterns for Motif' section of the Console.

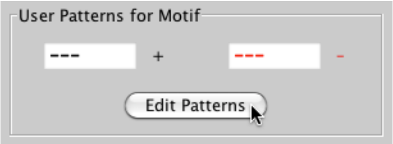

3. In the 'Edit Patterns' dialogue box select 'T' in the first drop-down list in the 'Specify Amino Acids' section (black) and 'G' in the second drop-down list in the 'Specify excluded Amino Acids' section (red). Then click 'OK'. The φψ plots should now display just 80 motifs, as shown below, and the panels in the 'User Patterns for Motif' section of the Console should have been updated to reflect the selected pattern.

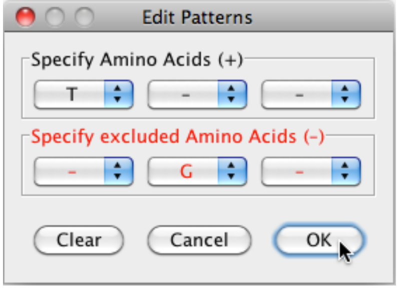

Nest: 1,2-αRαL (pattern: T--/-G-, 80 motifs)

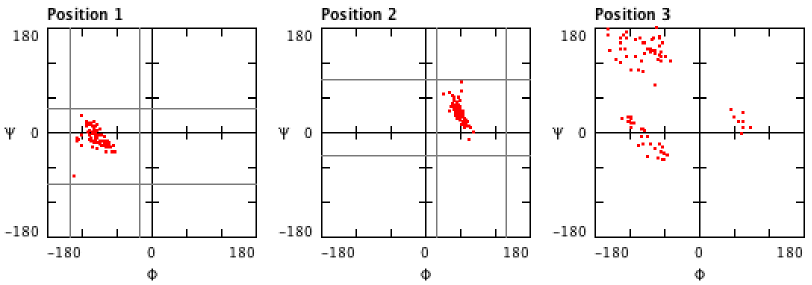

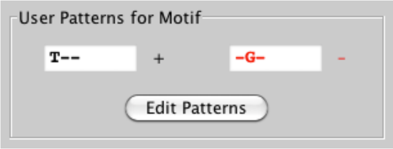

4. Let us suppose we wish to take a closer look at the motifs conforming to the pattern we have chosen. Select 'Inspect Motifs' from the 'Extras' menu. A window appears, as shown on the opposite page. (The motifs displayed by 'Inspect Motifs' are those from whatever selection has been made, either by pattern or by area or both. If there is no selection, all members of the current motif will be displayed.)

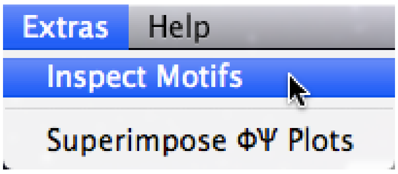

5. The list presents sufficient information for each motif that one can identify it after having launched the relevant protein file in the three-dimensional structure viewer of one's choice. Although *Structure Motivator* does not have its own 3D viewer, it provides links to a facility on the *Motivated Proteins* website that will display each motif with *Jmol* in the user's browser. This is convenient if one wishes to perform a rapid initial survey of the motifs listed.
6. To examine a motif in this way, select it in the list, and then click the 'View in Jmol' button. Your web browser should launch, and a representation of the backbone of the protein should appear with the motif highlighted in red, as shown below. If you click on the button for 'Backbone & Secondary Structure', the position of the motif is shown in relation to secondary structure (in this case at the *C*-terminus of an α-helix), as can be seen to the right.

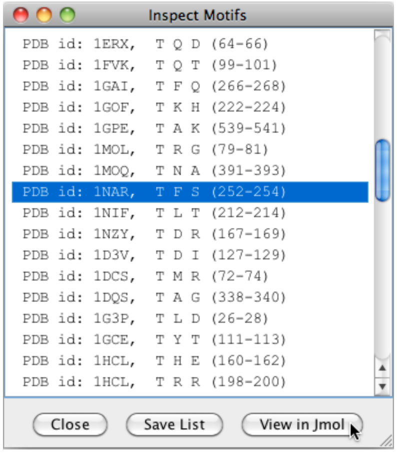

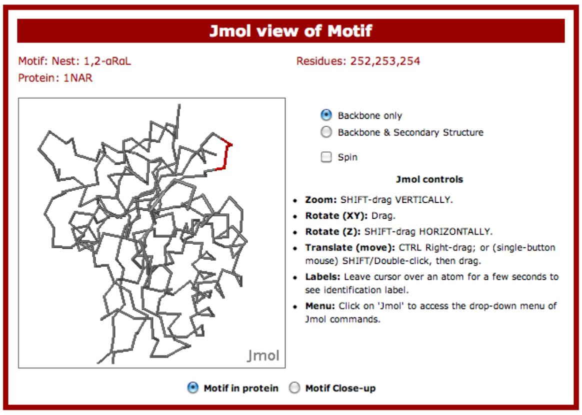

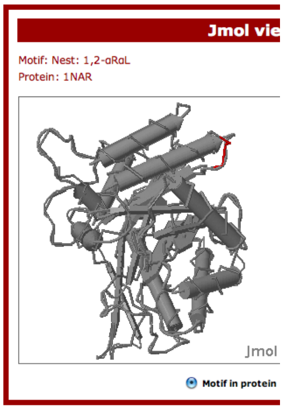

Superimposition and import

1. *Structure Motivator* allows one to superimpose the plots for the different residues of a motif in a single graph. We shall illustrate this with a motif that is not included in the embedded database, but is specified in one of the files available for download. (If for any reason you are unable to obtain this file, you can use the 'αRαR Beta Turn' instead. To follow the tutorial fully you should export the data first.)
2. Select 'Open File' from the 'File' menu, and load the file, 'alphaTurn.smo'. This file contains structural data for the 'pure' α-turns used in one of our papers (Proteins 2011; 79: 1010–19). On loading this (or any other) custom file, the motif window has the appearance opposite. The φψ plots for this motif set are shown overleaf.
3. Although the αR conformation is predominant in each of the first four residues of this motif, its location and distribution varies between them. Confirm this by allowing the mouse to rest at the 'north-west' position of residues 1 and 4 and reading the co-ordinates from the 'tool-tips' (shown overleaf).

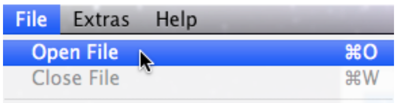

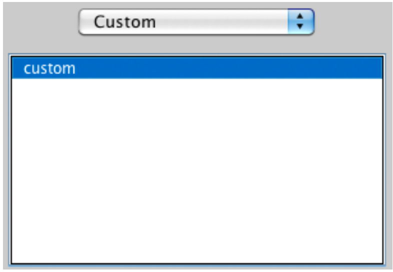

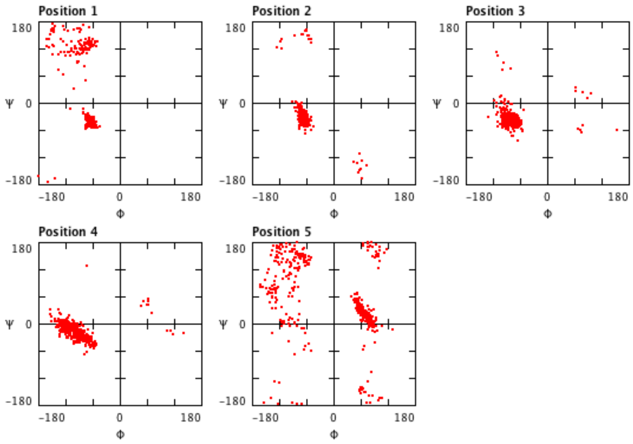

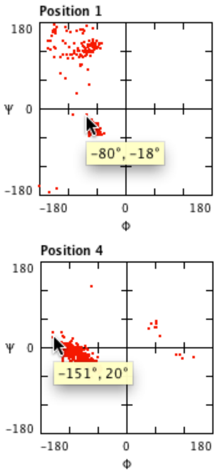

4. Select ‘Superimpose φψ Plots’ from the ‘Extras’ menu. The superimposition window appears with the five residue positions all been on the same graph, each in a different colour (shown below).

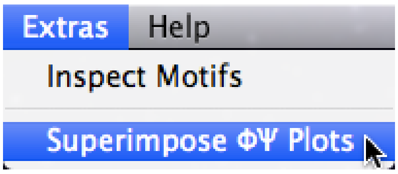

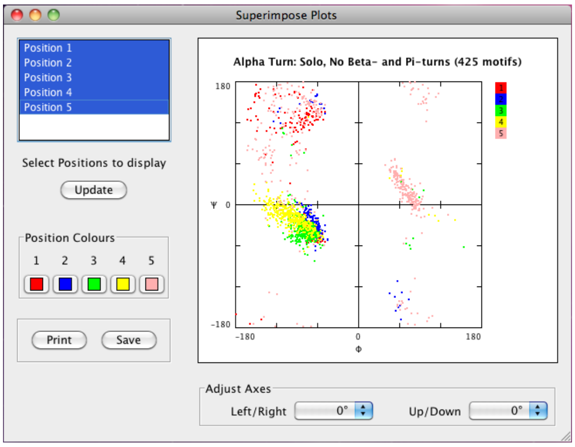

5. We would like to customize the display. We do not want to include position 5, so we deselect it and click ‘Update’.
6. We would prefer that the broader distribution of position 4 be drawn first, so that the other positions are not obscured. To do this we drag the positions into the order we want them drawn. We also decide to change the colours. After updating, the result is shown below, and can be saved or printed if required.
N.B. Before loading another file close the current one first.

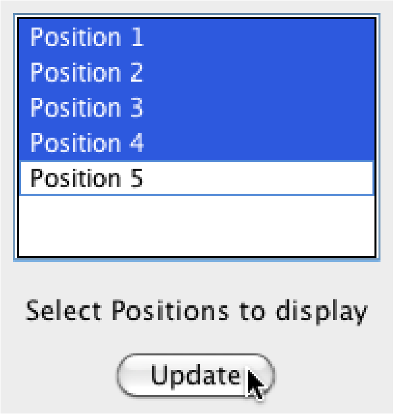

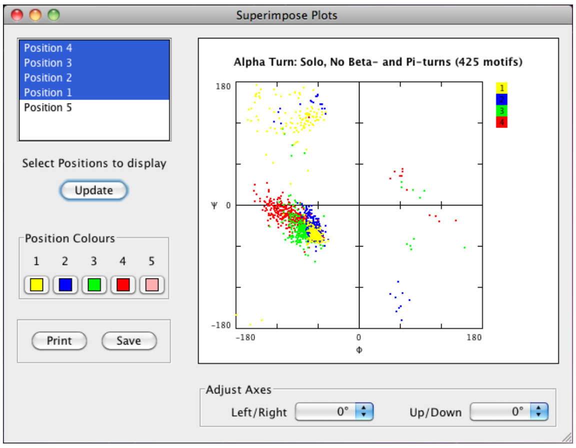

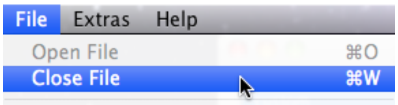

3. Menu and Console Reference

Menu Items

The *Structure Motivator* menu bar contains three menu items: ‘File’, ‘Extras’ and ‘Help’. This section provides a brief summary of each menu item.

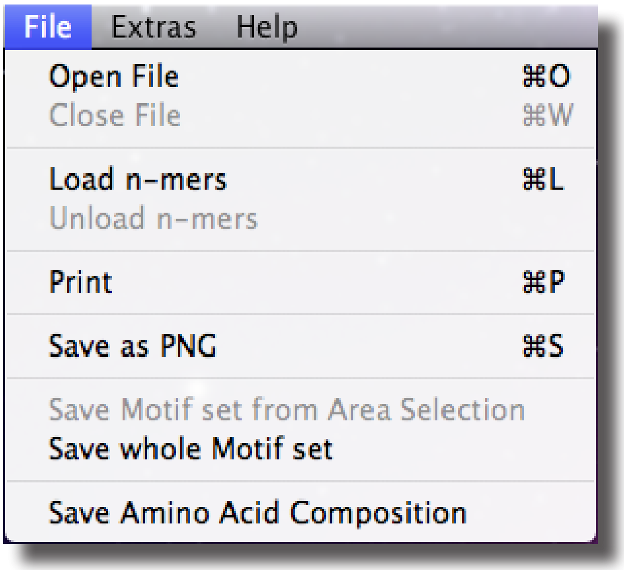

Open File (File menu)

This allows one to load an external file for viewing in *Structure Motivator*. In general the file format should conform to the tab-separated text output from ‘Save whole Motif Set’ (p. 12), but one should consult Section 4 for a complete description of the file format. Only one file may be loaded at a time, so this menu item will be dimmed until any currently loaded file is closed.

Close File (File menu)

This allows one to unload a previously loaded file from *Structure Motivator* in order to load another, as discussed above. This menu item is dimmed if there is no currently loaded file. (The reload operation is quicker if some relatively rare motif is selected before closing.)

Load n-mers (File menu)

This is an option for loading an internal file containing approx. 90,000 small peptides represented in the proteins in the embedded database. One is prompted to choose the length of peptide — from 3-mer to 6-mer. The purpose of this is to provide the user with an absolutely clean starting point from which to make interactive selections or specify sequence patterns. The proviso here is that one needs a machine with suitable processor to use this option to maximum advantage. Most machines will be able to load the data — after a short lag — but users of computers with older processors my find making interactive selections to be unsatisfactory. In the latter case the option (p. 15) of decreasing the number of points displayed to 10,000 may be worth considering.

Unload n-mers (File menu)

Only a single custom file — be it n-mer or external file — can be present in *Structure Motivator* at any time. So, for example, if one makes a selection from loaded n-mers and then saves a motif set (p. 12), it is necessary to unload the n-mers before one can reload the saved set for further manipulation.

Print (File menu)

This allows single-page printing of the displayed graphics. There is no ‘Print Preview’, so one needs to check that the page orientation is appropriate for a particular display, and that the overall layout is not too large for a single page.

The Print menu is also be used if one wishes to obtain publication-quality file output. This is done by printing to PDF — an integral option in Mac OS X, and available with third-party software in Windows.

Save as PNG (File menu)

This saves a copy of the displayed graphics in portable network graphics (PNG) format, which is suitable for web and screen use. As with printing, one should check that the page orientation is appropriate, and that the overall layout is not too large for a single page. PNG is not advised for publication-quality graphics, which are best obtained by printing to PDF (*see* 'Print', above).

Save Motif set from Area Selection (File menu)

This allows one to save data for motifs highlighted by marquee selection — *see* Console. (To save the data for all instances of the current motif, use 'Save whole Motif Set'.) The output is a text file in the format that is used for import (*see* above and Section 4), but it is also suitable for opening in a spreadsheet. (For *MS Excel*, import as tab-delimited text from row 5.)

Save whole Motif Set (File menu)

This is similar to the above, but includes all the instances of a particular motif, even if there is currently a marquee selection. However if a pattern has been specified, the exported set is restricted to motifs conforming to the pattern.

Save Amino Acid Composition (File menu)

Amino acid composition (%) for AlphaBeta Loop: 2,3-aRaR [2462 motifs]

	A	R	N	D	C	Q	E	G	H	I	L	K	M	F	P	S	T	W	Y	V
1	8	4	5	7	1	3	5	4	3	7	10	4	2	6	5	6	5	3	4	8
2	11	5	3	5	1	4	8	5	2	5	10	6	2	3	10	7	3	2	3	5
3	12	6	5	9	0	6	12	5	2	2	6	8	2	2	4	8	4	1	2	3
4	8	5	3	5	2	6	8	3	3	7	10	5	3	5	0	4	5	2	3	10
5	8	5	3	3	2	4	5	3	2	11	15	4	3	5	0	3	4	2	5	14

This allows one to save the percentage amino acid composition for each position in the motif to a tab-separated text file. The file (an example of which is shown below for a motif with five residues) is suitable for import into a spreadsheet and for subsequent graphing.

Inspect Motifs (Extras menu)

This brings up a window with a listing for each of the motifs in the current set (either all of them, if no selection has been made, or those selected by marquee or pattern). Individual motifs in the list can then be chosen and viewed in three-dimensions by double-clicking or using the 'View in Jmol' button. This launches the user's default web browser and loads an external web page containing the *Jmol* applet, configured to allow the motif to be viewed either in the context of the protein subunit or in isolation.

This *Jmol* visualization facility only operates for external files with motifs from the approx. 500 protein subunits in the database.

It is also possible to save the listing to a text file for later use.

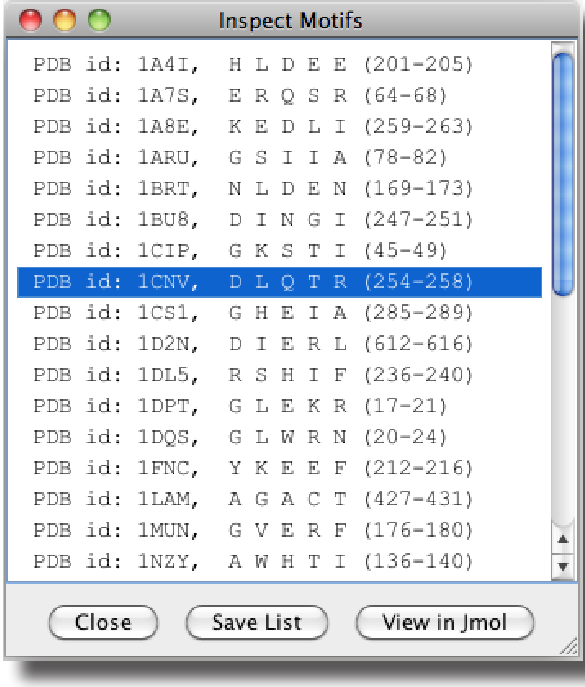

Superimpose φψ Plots (Extras menu)

This brings up a window in which the different residue positions of a motif sub-category are drawn on a single φψ plot, colour being used to distinguish them. The positions in the motif are listed, and only those selected are displayed. Initially this is all of them, but positions can be deselected to restrict display to those of interest. The positions are drawn in the order they appear on the listing, but the order can be altered by dragging. Thus, in the illustration opposite display has been restricted to positions 2 and 3, and their order has been changed to illustrate better the relative areas of the points. The colours may be customized, and the graph axes adjusted, if desired.

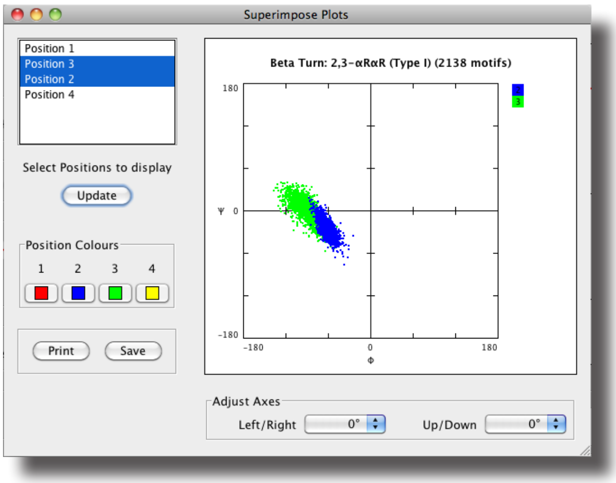

Help menu

This provides access to four in-built Help topics and two external Help files. The in-built Help provides an Introduction to the application, a description of the Console controls, a description of the Menu controls, and the options that are available by different sorts of Mouse actions within individual graphs. The menu item, ‘Motif Glossary’, provides a link to a set of web pages with detailed descriptions of the motifs in the database. These should launch in the user’s web browser, and may resize automatically, depending on the browser and whether or not it is already open. Finally there is a link to the *Structure Motivator* website, from where the latest versions of the application, this manual and the additional files can be downloaded.

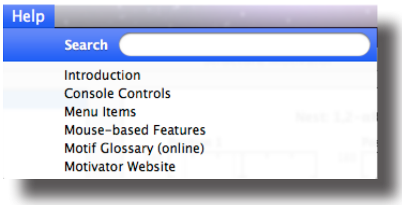

Preferences

The Preferences menu item is found in the ‘Motivator’ menu of Mac OS X, and in the ‘Help’ menu of Windows. It invokes a dialogue box which is currently limited to settings for Colour Preferences. One may change the plot colour and selection colour for the graphs from their default red and blue, respectively, in a pop-up menu invoked by clicking on the current colour. (If the same colour is selected for both plot and selection, the application will revert to the default colours.)

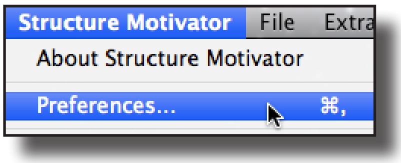

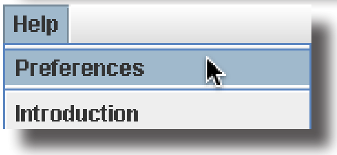

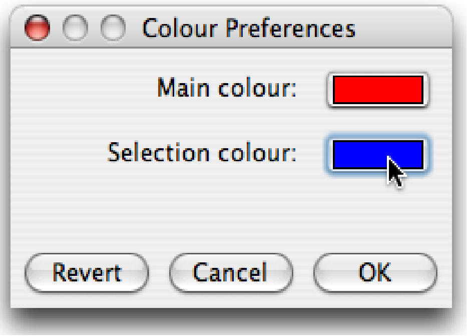

The Console (located to the left of and beneath the graphical display canvas) presents the user with controls for the main options in *Structure Motivator*. This section provides a brief summary of each control, starting with those on the left (shown opposite) working from top to bottom, followed by the section beneath the display (shown on the next page).

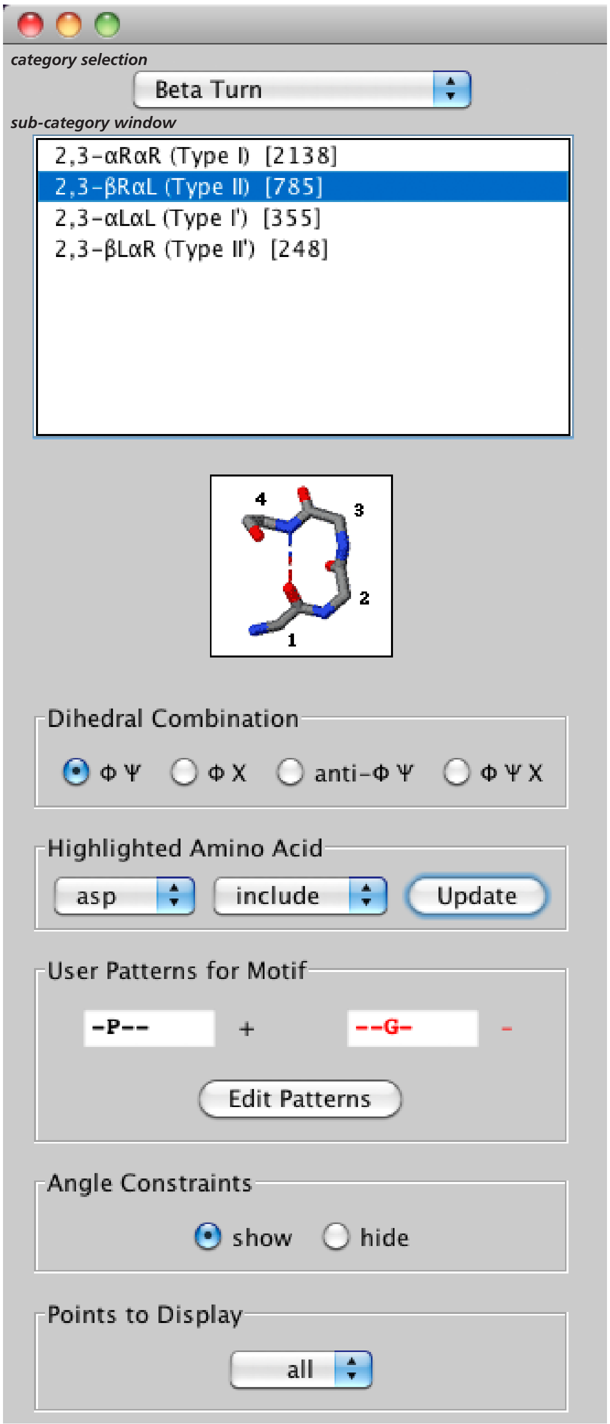

Motif category selection and sub-category window

The name of the current motif is shown on the category-selection drop-down menu, with sub-categories of the motif in the window listed beneath it. The number of examples of a particular sub-category in the database is indicated in square brackets. On selecting a different category from the menu, or on clicking a new sub-category, the graphic display updates automatically.

Cartoons

Below the category selection menu is a small graphic frame in which cartoon representations of motifs are presented. They show any hydrogen-bonding constraints that define the motif, and the numbering of the positions corresponds to that used in the graphs. The current cartoon represents the sub-category selected in the list, although if this is a custom motif the cartoon will be blank.

Dihedral Combination

This section of the Console provides the user with four options. The first ($\phi\psi$), the default, is for standard Ramachandran plots comparing ϕ and ψ angles at each amino acid residue in a motif. The second ($\phi\chi$) allows one to view $\chi 1$ angles, which, to allow better visualization, are (somewhat arbitrarily) plotted against ϕ angles. Next, there is the option for what we term anti-Ramachandran plots (anti-$\phi\psi$). Here the ϕ angle for one position in a motif is plotted against the ψ angle in the following position, i.e. the graphs display the dihedral angles flanking the peptide bonds, rather than those for the α-carbons of residues. A final option, $\phi\psi\chi$, allows one to view $\phi\psi$ and $\phi\chi$ plots together. Individual graphs are obviously smaller in this case than in the individual $\phi\psi$ and $\phi\chi$ plots, but the mode allows one to see which ϕ and ψ angles correspond to the selection (*see* below) of a particular set of $\chi 1$ angles, and vice versa.

Highlighted Amino Acid

This section of the Console contains two drop-down menus and an ‘Update’ button. The first menu allows one to select a particular amino acid. Having selected an amino acid one has three choices in the second menu: ‘include’ means that dihedral angles for the chosen amino acid will be included but will be drawn last and highlighted (in blue), ‘exclude’ means that dihedral angles for the chosen amino acid will be excluded from the display, and ‘sole’ means that only dihedral angles for the chosen amino acid will be displayed. The choice is implemented by clicking the ‘Update’ button. It is important to realize that the highlighted (or excluded) points reflect the occurrence of the particular amino at different positions in all instances of the current motif, rather than representing a set of instances of the motif, which is the case when a pattern is selected (below).

This option is not available when ‘anti-$\phi\psi$’ is selected in the ‘Dihedral Combination’ section (above). It is negated if one clicks on one of the ‘Area Selection’ tools (below).

User Patterns for Motif

This option allows one to work with only those motifs conforming to a particular pattern of amino acids. One can specify both amino acids that must be present at particular positions in the sequence (displayed in black) and amino acids that must be absent (displayed in red). To specify a pattern one clicks on ‘Edit Patterns’, makes selections in the dialogue box that appears, and then clicks ‘OK’. The choice is then registered in the display and the graphs are updated to reflect the selection.

The selection of amino acids in this option contrasts with the amino acid highlighting (above) in that here a subset of instances of the current motif is selected. Indeed, one can still highlight a particular amino acid after setting a pattern.

Angle Constraints

The definitions of some of the motifs involve constraining the dihedral angles to a particular range of values at certain positions. By default these constraints are shown as grey boundary lines, so that the user is aware that points falling in the constrained regions do so by deliberate selection. The grey lines can be hidden, if desired (e.g. for printing), by clicking the ‘hide’ button.

Points to Display

This option allows one to display a proportion of the points, rather than all of them. It may be useful in conjunction with the more abundant motifs, where plots of all of the points may give a misleading idea of their overall distribution.

Area Selection

This section of the Console contains buttons for the two shapes of selection tool, a tick box for drawing a straight line through a selection of points, and a rectangular window for displaying the associated statistics. To activate the tool for making an elliptical or rectangular selection of points one clicks on the button with the corresponding graphic. Selections are made by dragging with the cursor, and can begin outside a square, but must finish within a square. The points for the selected instances of a motif are highlighted in blue, both on the graph of the motif position in which the

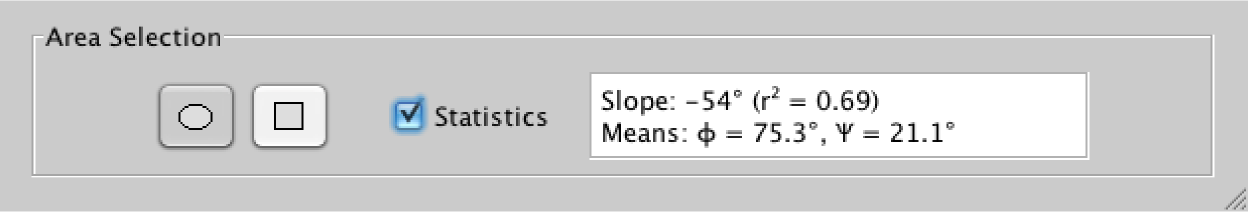

selection was made, and in the graphs of the other positions of the motif. The number of points selected is indicated in the text box. It is sometimes useful to be able to make an 'inverse selection', i.e. to cause all the points *outside* the 'marquee' area to be selected and to exclude those within it. This is done by holding the 'alt' key down while creating the selection area.

If φψ has been selected as the dihedral pair and the box labelled 'Statistics' is ticked, a least-squares regression line will be drawn through the selected points, and the angle and statistic r^2 displayed in the text box (instead of the number of points selected). The nearer to a value of 1 that r^2 is, the better is the fit of the line. However users should inspect their results carefully, as least-squares regression may not be appropriate for a particular selection of points. If φχ has been selected as the dihedral pair, the best horizontal mean line through the points is drawn. Statistics are not available in φψχ mode. The text in the statistics window is selectable and may be copied and pasted into a text file.

When the area-selection tool is active any amino acid selection that had previously been made is over-ridden. To exit 'Area Selection', click again on the button for the active selection tool to deselect it.

Mouse-based Features

This section summarizes those operations that pertain to individual graphs, and are therefore initiated by mouse actions within the graph in question.

Adjusting Graph Axes

Double-clicking within a graph invokes a dialogue box that allows one to shift the *x* or *y* axes for that graph in increments of 30°. This can be useful when points are in a cluster, but fall at either side of one of the default limits (+180° or –180°) normally used in Ramachandran plots. The graphs for other positions in the motif remain unaffected and can be adjusted independently. This facility is not available for the φχ plot of the φψχ mode.

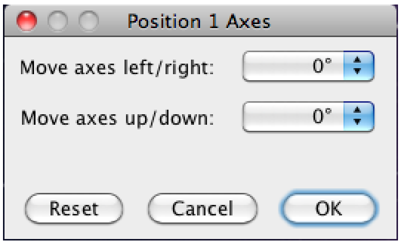

Amino acid Composition

Right-clicking (control-clicking, for a single-button mouse) within a graph brings up a list showing the amino-acid composition at the position in the motif to which the graph corresponds. This may be a useful preliminary to visualizing different amino acids with the amino acid highlighting option or to specifying a particular pattern.

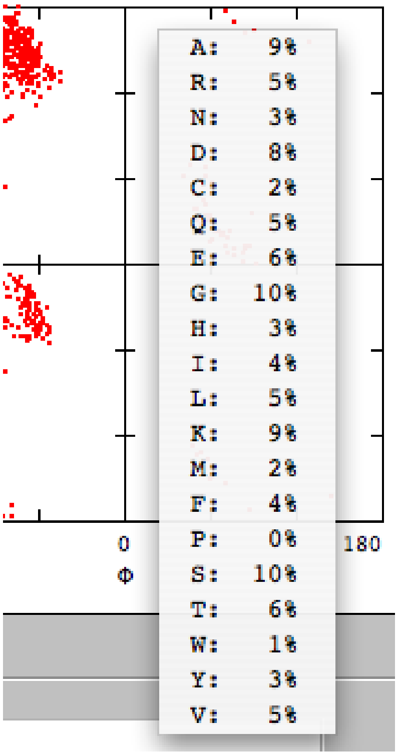

Graph Co-ordinates

Allowing the mouse pointer to rest over any of the graphs for a few seconds causes the co-ordinates at the tip of the pointer to be displayed.

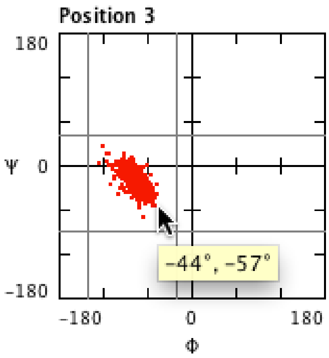

4. File Format for Import and Export

As described elsewhere, *Structure Motivator* can display external data imported from files in a format which is identical to that in which data is exported. This format is based on plain text with tab-separated fields, and one can familiarize oneself with it by examining the files of additional motifs that are available for download from the *Motivated Proteins* website. (These have the extension 'smo', which may be confusing as exported files have the extension 'txt'. In fact text files with any or no extension are accepted by *Structure Motivator*, provided that they are correctly formatted.)

Part of one of the files from the website ('Catgrip23bLbR.smo') is shown below. It consists of a 'header' section followed by the data, with that for each motif being on a separate line.

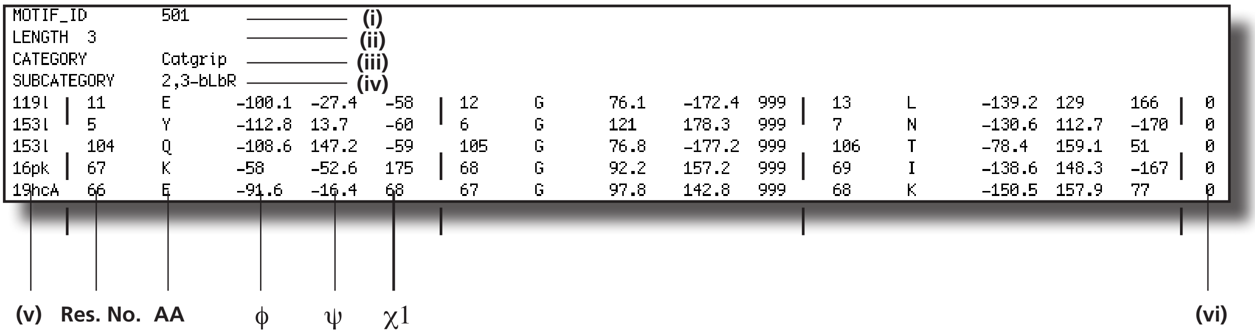

Header

(i) The first line starts with the word MOTIF_ID, separated by a tab from a number. For motifs in the database this is an identification number from 100 to 200, and determines the cartoon graphic displayed. When importing data that does not correspond to a sub-category in the database one must still specify a numerical value, although this can be quite arbitrary.

(ii) The second line starts with the word LENGTH, separated by a tab from a number which is the length (number of residues) in the motif. This is obligatory, and data for motifs of mixed length cannot be imported.

(iii) The third line starts with the word CATEGORY, separated by a tab from a textual description of the motif category, in which spaces are allowed. The entry on the CATEGORY line will appear in the title of the graph display, but need not correspond to any motif in the database.

(iv) The fourth line starts with the word SUBCATEGORY, separated by a tab from a textual description of the motif sub-category, in which spaces are allowed. If this in inapplicable, insert 'none' or a dash. This will also appear in the title of the graph display.

Data Lines

The data lines contain tab-separated fields as follows:

(v) The first item on each data line is a protein ID. This need not actually conform to the four-character PDB format, but an entry in this field is obligatory.

(vi) The last item on each data line shown here is a motif ID. It is *not* obligatory, nor need it be unique. It is included mainly for the developer's convenience to allow exported motifs to be correlated to those in the original MySQL database from which the data in *Structure Motivator* originates (*see* Appendix I).

For each position in the motif there is an entry for residue number, amino acid (single-letter, upper-case), and values for the ϕ, ψ and $\chi 1$ dihedral angles, all separated by tabs. Absent or unknown angles should be given the value 999, and unknown amino acids the value 'X'.

Appendix I: The Database: Proteins and Motifs

Structure Motivator accesses a database of 19 categories of protein motif, with 100 potential sub-categories, drawn from over 400 proteins from the Richardson 'Top 500'.

Details of the database are available on the *Motivated Proteins* website (http://motif.gla.ac.uk/index.html) and in a publication (*BMC Bioinformatics* 2009, **10**:60).

Details of the motifs can be found in an on-line glossary (http://motif.gla.ac.uk/motifhelp/index.html), also linked from the Help menu. When working in *Structure Motivator* the angle constraints are indicated in the plots, and the hydrogen bonding definitions are indicated in the cartoons of individual motif sub-categories.

The embedded Java database can be found in the 'Motivator.jar' file in the Unix/Linux version of *Structure Motivator* and can easily be extracted from this by technical users. It constitutes that part of the full MySQL *Protein Motif* database underlying *Motivated Proteins* that is highlighted in the diagram below. (Non-technical users can export the data for each motif sub-category in a form that can easily be imported into spreadsheets.)

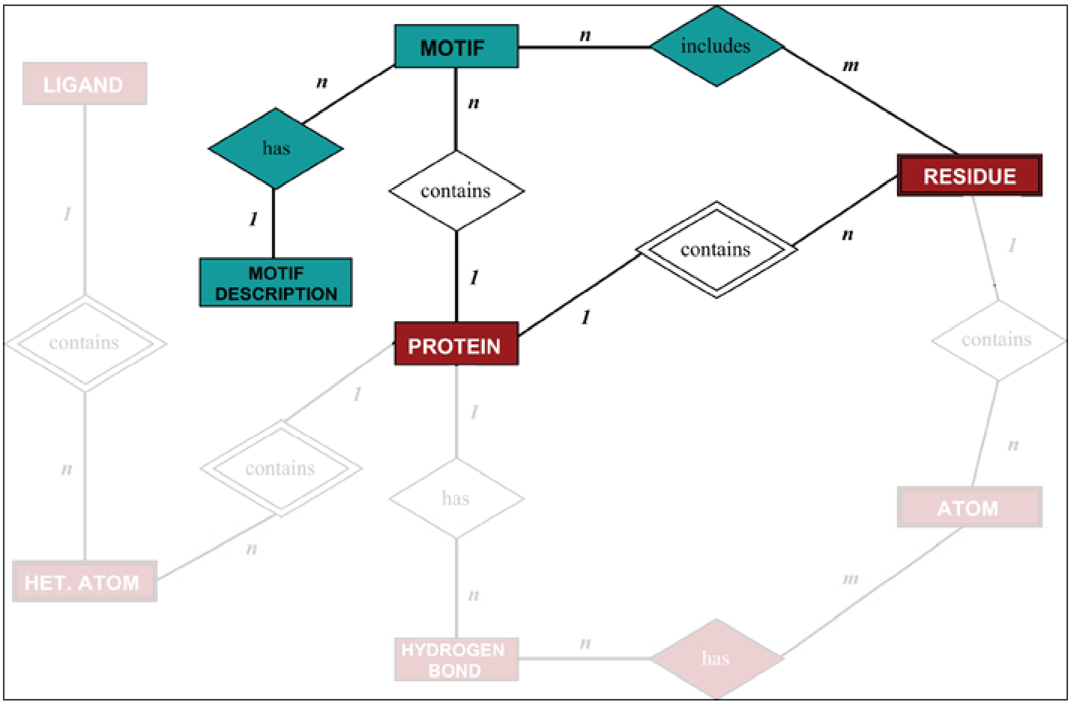

Appendix II: Statistics

To calculate the best straight line through a cluster of points *Structure Motivator* uses linear regression, employing the *Apache Commons Math* Java statistics library (http://commons.apache.org/math/). Linear regression requires one to designate one variable as dependent and the other as independent. For $\phi\psi$ plots there is no obvious dependent variable, but a different value is obtained for the slope of the curve depending on whether one chooses ϕ or ψ. The program therefore runs the regression with both possibilities and displays the one with the lowest 'Error Sum of Squares'. This is useful in that it seems to give reasonable values in many cases. However the user who requires rigorous values is advised to export the data from *Structure Motivator* and analyse it with a statistics package that implements Principal Component Analysis (not apparently currently available for Java).

Appendix III: *PreMotivator*

PreMotivator is a small utility for preparing files of user-specified motifs suitable for direct input into *Structure Motivator*. The user loads a simple text file specifying a pattern of main-chain dihedral angles (ϕ and ψ) for each residue of a short peptide (up to 9 residues in length), with the option of allowing the conformation at particular positions to be unrestricted. After loading the file, the desired range for the angles (±10°–60°) is selected and the utility run. A search is made for matching instances of the pattern in the same embedded database that is included in *Structure Motivator*. The results are displayed, giving one the opportunity to change the stringency if desired, and they may be exported as plain text files with the 'smo' extension. These files are suitable for immediate loading into *Structure Motivator*, although they may be edited if required (e.g. to change the descriptive heading).

The input files for *PreMotivator* should be in plain text format, with the ϕ and ψ values for each residue written on a separate line and separated by a tab. The last line of text should be followed by a carriage return. Where the user does not wish to constrain the angles at a particular residue, the value 999 should be specified. An example is shown below for tetrapeptides in which residues 1 and 3 are in the αL conformation, whereas those at residues 2 and 4 are not defined.

```
55	37
999	999
55	37
999	999
```
